# Luminescence and Palladium: The Odd Couple

**DOI:** 10.3390/molecules28062663

**Published:** 2023-03-15

**Authors:** David Dalmau, Esteban P. Urriolabeitia

**Affiliations:** Instituto de Síntesis Química y Catálisis Homogénea (ISQCH), CSIC-Universidad de Zaragoza, Pedro Cerbuna 12, 50009 Zaragoza, Spain; ddalmau@unizar.es

**Keywords:** fluorescence, palladium, orthometallation, porphyrins, AIE, TADF, metallacages

## Abstract

The synthesis, photophysical properties, and applications of highly fluorescent and phosphorescent palladium complexes are reviewed, covering the period 2018–2022. Despite the fact that the Pd atom appears closely related with an efficient quenching of the fluorescence of different molecules, different synthetic strategies have been recently optimized to achieve the preservation and even the amplification of the luminescent properties of several fluorophores after Pd incorporation. Beyond classical methodologies such as orthopalladation or the use of highly emissive ligands as porphyrins and related systems (for instance, biladiene), new concepts such as AIE (Aggregation Induced Emission) in metallacages or in coordination-driven supramolecular compounds (CDS) by restriction of intramolecular motions (RIM), or complexes showing TADF (Thermally Activated Delayed Fluorescence), are here described and analysed. Without pretending to be comprehensive, selected examples of applications in areas such as the fabrication of lighting devices, biological markers, photodynamic therapy, or oxygen sensing are also here reported.

## 1. Introduction

The synthesis and custom design of luminescent compounds has experienced an exponential growth during the last decades, which still continue today. A nice proof of the present interest in the area is the fact that the IUPAC has included three technologies related to and/or derived from luminescent compounds in 2020, 2021 and 2022 in the “Top Ten Emerging Technologies in Chemistry”: aggregation-induced emission (AIE) in 2020, chemoluminescence in 2021 and fluorescent sensors in 2022 [1,2,3]. This is due to the high added value of these smart materials and the wide variety of applications they show. Just to highlight the most important, we can find luminescent materials in fields with scope as wide as sensors [4], image biomarkers [5,6,7,8,9,10], light-emitting devices (OLEDs) [11,12,13,14], photodynamic therapy [15] or theragnosis [16,17,18,19,20], and also in more specific areas such as host–guest encapsulated materials [21], among others.

Luminescent materials can be of organic or organometallic nature, each group having their own advantages and drawbacks. Organometallic complexes belonging to the second and third rows (Ir, Pt, Au, Ru, Re) have been intensely developed due to their intrinsic properties. The presence of a transition metal with a high spin–orbit coupling constant in such organometallic complexes allows the possibility of Intersystem Crossing (ISC) [22,23,24], more likely the higher the constant is, allowing the population of the lowest energy triplet state T_1_ from the lowest energy singlet S_1_, therefore promoting phosphorescence emissions in addition to the expected fluorescent pathways. This drives to an optimum harvesting of excitons because both singlet and triplet excitons are involved in light generation, increasing notably the intensity of the emission [25,26]. This has been one of the main reasons for the success and exploitation of the photophysical properties of metallic complexes, although a series of additional considerations have to be remarked in this point.

**Figure 1:**
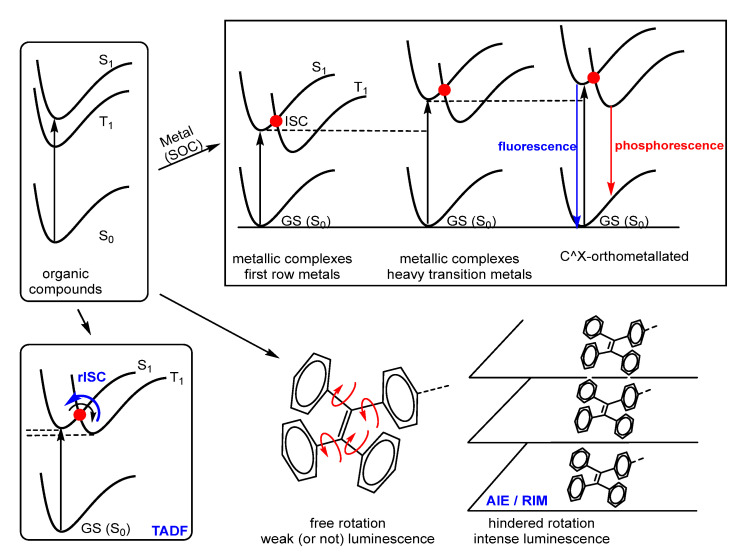
Schematic representation of the old and new concepts presented to recover and amplify the luminescence of transition metal complexes. Adapted from references [27,28] (complexes of the first row metals), [29,30,31,32,33,34,35] (orthometallation), [36,37] (RIM), [38,39,40] (AIE) and [41,42,43,44] (TADF).

The introduction of the metal orbitals in the molecular set of orbitals play additional roles in the final properties of the metal luminophores. For instance, in the 3d metals of the first transition row, the weak crystal field promotes small d-d splitting, much smaller than those of their corresponding 4d or 5d neighbours, because 3d orbitals are more compact and show a less efficient overlap with the orbitals of the ligands than in the case of 4d or 5d orbitals. Therefore, for 3d metal complexes, there are many excited states centred in the metal (MC) quite close in energy among them, and also with respect to the ground state, allowing an easy population of all levels (Figure 1). This promotes easy and fast metal-centred non-radiative relaxation processes, which finish in the ground state, deactivating any possible radiative decay. This is one of the main reasons by which luminescent 3d organometallic complexes are scarcely represented even at the present time [27,28].

The higher energetic gap found in the case of 4d and 5d complexes due to the better metal–ligand overlap of orbitals, not only between them but also with respect to the ground state, makes non radiative relaxations less efficient and, as a consequence, fluorescence and/or phosphorescence become more intense. On this point, the role of the ligands to further improve the performance of the photophysical properties is critical. A well established methodology to achieve this task is the use of strong-field ligands mainly containing C and N as donor atoms, although P and S are also used in lower extent (Figure 1). Cyclometallated ligands are by far the type of ligands most extensively used for this purpose due to several reasons [29,30,31,32,33,34,35]. The main one is that they promote very large splittings of the crystal field, raising non-emissive d-d MC states and further decreasing non-radiative relaxation. In addition, the chelating nature of the cyclometallated ligands allows a better thermodynamic stabilization of the resulting complexes and, moreover, the synthesis of the ligands is highly modular, allowing the tailored introduction of substituents with different requirements at specific positions of the ligands. In turn, this enables the fine control and modification of the photophysical properties. More often than not, the use of cyclometallated ligands is combined with rigid structures, in such a way that the luminescent properties are amplified due to the Restriction of Intramolecular Motions (RIM) [36,37]. Both intramolecular rotational and vibrational motions are also a typical source of deactivation, and the molecular design of new fluorophores usually consider their elimination, as much as possible, to get the best results in terms of quantum yield.

Palladium is a 4d metal for which one should expect, in principle, a splitting of the d orbitals large enough to make radiative emissions at least competitive with non-radiative relaxation. In addition, cyclopalladated complexes having more or less rigid structures are probably one of the most developed families of Pd complexes. Despite these facts, the experimental results show that Pd is the ugly duck of the luminescent compounds among the 4d and 5d transition metals, and there are very few Pd complexes with remarkable photophysical properties. That is, the presence of Pd in organometallic complexes and the simultaneous observation of strong luminescence is still a strange feature, even if the same concepts and methodological considerations as in other metals have been taken into account. In fact, palladium is often used to quench the fluorescence of the ligands it bonds, this finally resulting in applications in sensors [45,46,47,48,49,50] or in the study of the intercalation of Pd complexes with DNA [51,52,53]. This quenching arises from the reasons above explained, the main one being the existence of MC (d-d) transitions easily available in the excited state, which open non-radiative relaxations.

Recent development of the area has shown that the use of cyclopalladated ligands [54] is not the only synthetic and methodologic tool to improve the luminescence of metallic complexes in general, as well as Pd derivatives in particular. The aggregation-induced-emission (AIE) [38,39,40], in combination with the building of supramolecular coordination complexes or metallacages [55,56], or the recent development of compounds with thermally activated delayed fluorescence (TADF) or metal assisted delayed fluorescence (MADF) [41,42,43,44], are new approximations from the achievement of highly luminescent platforms containing palladium.

Molecules having AIE (aggregation-induced emission) experience an increase of the luminescence as their concentration (aggregation) increases, a concept opposed to the well-known ACQ (aggregation-caused quenching) usually observed in most of the photoluminescent compounds. Following that, a given compound non-luminescent in dilute solution, but strongly emissive in concentrated solutions and in solid state, is called an AIEgen. This feature was observed years ago, but the mechanisms proposed were, in the best cases, ambiguous. In 2001, Tang, using silole compounds (non-luminescent in solution and highly emissive as nanoscopic aggregates), studied the phenomenon and disclosed that the restriction of intramolecular motions (RIM) caused by the aggregation [36,37] opened fluorescent channels for energy release from an excited state and that this was the general mechanism underpinning AIE [38,39,40]. Thermally Activated Delayed Fluorescence (TADF) is another still new concept, scarcely developed and not fully understood. This phenomenon happens usually when the difference of energy between the singlet excited state S_1_ and triplet excited state T_1_ is quite small. After excitation and population of the S_1_ level, ISC takes place with concomitant population of the triplet state T_1_, harvesting all possible excitons. Since the difference of energy between S_1_ and T_1_ is small, thermal energy provided by the surroundings of our molecule can promote a reverse intersystem crossing and singlet state S_1_ is re-populated. Then, radiative relaxation occurs, and fluorescence is observed. The difference is that after TADF, the 100% of the possible excitons have been formed, and therefore, the intensity of the fluorescence arising from TADF is much higher than that resulting from conventional relaxation [41,42,43,44].

This contribution reviews the most important experimental and conceptual advances produced in the period 2018–2022 in the design and synthesis of palladium complexes with intense fluorescent properties. The organization of the review comprises a first part devoted to the new contributions in classical orthopalladated systems, followed by most impressive reports in metallacages and supramolecular complexes. Next, the chemistry of the always surprising porphyrins and porphyrin-like ligands will be presented. In most of the cases, an amplification of the photophysical properties from a weakly luminescent ligand to a bright metallic luminophore in solution is observed. Although the number of Pd complexes with strong luminescence is still low in comparison with the Ir, Pt, or Au counterparts, the new Pd structures here described show how these advances pave the way for future discoveries and improved applications in energy, image, or health.

## 2. Orthopalladated Ligands

Compared to highly luminescent Ir(III), Pt(II), and Au(III) complexes, Pd(II) organometallic complexes typically exhibit a much weaker spin–orbit coupling (SOC) effect and lack an efficient radiative decay process from T_1_ → S_0_ [57]. Additionally, the Pd(II) complexes usually show thermally accessible metal-centred (MC) (d-d*) states, resulting in excited-state structure distortion and effective nonradiative decay [58]. Using strong sigma donor ligands, such as metallated ligands, is a common strategy to promote luminescence in transition metal complexes by raising the energy of thermally accessible d-d* states. However, this approach is not always effective. Despite these challenges, researchers have made significant efforts in designing and using organometallic palladium complexes as luminescent agents, particularly for their potential use in red-shifted emitters and OLEDs. We will categorize our analysis according to the type of ligands bound to the palladium centre to better understand the underlying mechanisms at play.

### 2.1. Monodentate Ligands

**Figure 2:**
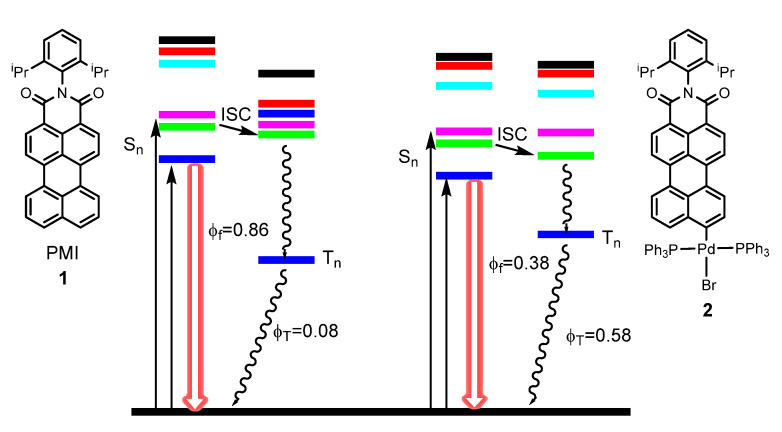
Jablonski diagram depicting the energy levels and the photophysics of PMI **1** and PMI-Pd **2**. Adapted from [59].

Hariharan et al. [59] studied how the incorporation of Pd affected the perylenemonoimide (PMI) chromophore **1**. They found that the incorporation of Pd was correlated with better access to triplet-excited states by ISC, associated with a greater spin–orbit coupling (SOC). However, the improvement of ISC with respect to the free ligand is not accompanied by the presence of phosphorescence. Moreover, the quantum yields of fluorescence drops going from 0.86 in the ligand to 0.38 in the Pd complex **2** (Figure 2).

### 2.2. Bidentate Ligands

**Figure 3:**
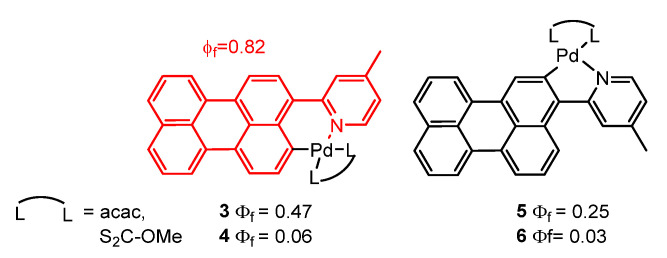
Fluorescence quantum yields of five and six-membered palladacycles. Adapted from [60].

Espinet et al. [60] studied the orthometallation of a perylenylpyridine ligand. They found that the reaction of the 2-(3-perylenyl)-4-methylpyridine (red colour) with Pd(OAc)_2_ yielded either five or six-membered metallacycles **3**–**6**, depending on the conditions used, as it is represented in Figure 3. The photophysical characteristics of the resulting metallacycles varied greatly depending on the type of cycle formed and the auxiliary ligand used. Five-membered palladacycles **5**,**6** exhibited coplanar perylene/metal-coordination-plane and perylene/pyridine arrangements, which led to lower fluorescence quantum yields compared to non-coplanar six-membered metallacycles **3**,**4**. The fluorescence quenching associated with planarity has been attributed to a photoinduced intramolecular electron transfer (PET) process induced by the pyridine. Additionally, acetylacetonate (acac) derivatives **3**,**5** were found to be 10 times more fluorescent than corresponding xanthate (S_2_C-OMe) compounds **4**,**6**, indicating a significant influence of the auxiliary ligand. The starting perylenylpyridine ligand showed a quantum yield of 0.82 in CHCl_3_ at room temperature, while the best results for the Pd organometallic complexes were 0.47 for **3** and 0.25 for **5**, both containing the acac ligand.

**Figure 4:**
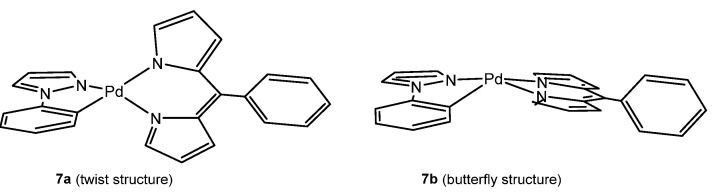
Twist and butterfly conformers of the Pd complex. Adapted from [61].

Marian et al. [61] investigated the photophysical properties of Pd(II) (phenylpyrazole) (phenyldipyrrin) complexes **7**. They found that the computed photophysical properties of the Pd complex vary depending on the structure it adopts, which are reported to be either twisted (**7a**) or butterfly-like (**7b**), as shown in Figure 4. However, there is low or no fluorescence, nor any phosphorescence emissions at all, regardless of the structure. The optimization of the S_1_ state yielded a dipyrrin intraligand charge transfer (ILCT) state with a highly distorted nuclear arrangement in the butterfly conformer, resulting in non-radiative deactivation. In the twist conformer, the presence of low-lying MC states leads to non-radiative deactivation of these emissions.

**Figure 5:**
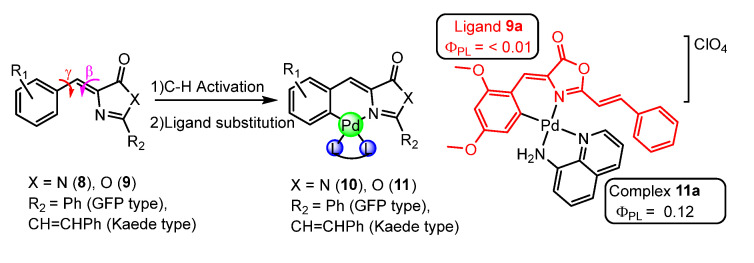
Amplification of the fluorescence of GFP-like fluorophores using Pd as an intramolecular lock. Adapted from [62,63,64].

Urriolabeitia et al. [62,63,64] made significant progress in developing luminescent palladium organometallic compounds using bidentate ligands. The ligands employed are fluorophores related to Green Fluorescent Protein (GFP) and Kaede protein **8**,**9**. One major challenge with these ligands is the “hula-twist” mechanism, a combination of γ+β rotations, which can lead to non-radiative deactivation of the excited state (Figure 5). To overcome this issue, they employed intramolecular anchoring to create six-member metallacycles **10**,**11** (Figure 5). Interestingly, the incorporation of palladium in these ligands, which showed itself to be non-luminescent in solution, led to the emergence of luminescence. The best results were obtained with ligands related to Kaede protein using aminoquinoline as an auxiliary ligand (**11a**) [63], where the quantum yield in solution at room temperature increased from less than 1% for the oxazolone to 12% for the orthopalladated complex.

### 2.3. Tridentate Ligands

**Figure 6:**
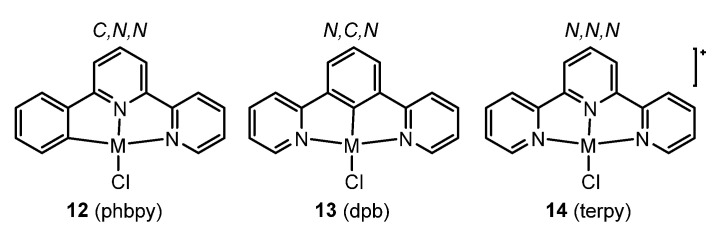
Neutral complexes with cyclometalating C,N,N and N,C,N coordinating ligands. phbpy and dpb compared with the analogous cationic complexes with the N,N,N coordinating terpy.

Cyclometalating tridentate or tetradentate ligands have shown to be highly suitable to create a strong ligand field and push the so-called “dark” (non-radiative) d-d* states in energy above the triplet-emitting metal-to-ligand charge transfer (MLCT) or π-π* states (or mixtures thereof). Amongst them, tridentate C,N,N (**12**) or N,C,N (**13**) coordinating units (Figure 6) derived from the prototypical tridentate N,N,N ligand 2,2′:6′,2′′-terpyridine (**14**) constitute a well-studied group.

**Figure 7:**
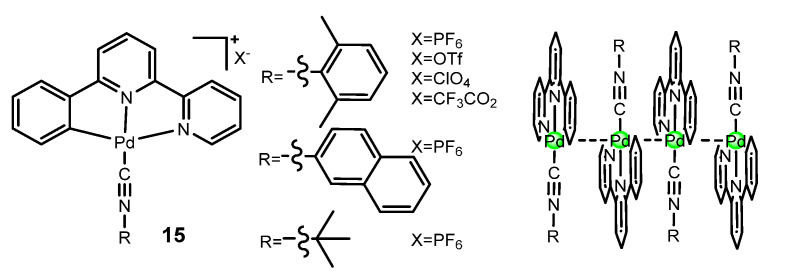
Chemical structures of the Pd(II) complexes and 1D infinite structure with a Pd(II)–Pd(II) chain with M–M interactions. Adapted from [66].

The incorporation of metal–metal interactions into excited states, such as the ^3^MMLCT (metal–metal-to-ligand charge transfer), can facilitate the radiative decay of the triplet excited state to the ground state due to the increased metal percentage in the frontier molecular orbitals. Essentially, bringing the two metal centres in close proximity allows for the overlap of the dz^2^–dz^2^ orbitals, which results in the creation of dσ and dσ* orbitals. This leads to the emergence of metal–metal-to-ligand charge transfer (MMLCT) transitions at a lower energy level compared to the mononuclear complex. The energy of the dσ* → π* MMLCT transition is largely determined by the degree of dz^2^ overlap and decreases as the metal centres come closer together, which is why it depends strongly on the metal–metal distance [65]. In recent decades, significant efforts have been made to uncover Pd(II)–Pd(II) interactions and metal–metal bonded excited states of luminescent Pd(II) complexes. Spectroscopic evidence for MMLCT excited states attributed to Pd(II)–Pd(II) interactions were first characterized by Che et al. [66] using the tridentate ligand phbpy in conjunction with different isocyanide ligands (Figure 7). They provide convincing evidence that pincer Pd(II) isocyanide complexes **15** exhibit intense ^1^MMLCT absorption and emissive ^3^MMLCT excited states upon supramolecular polymerization. Quantum yields above 0.4 were obtained at room temperature for the aggregates.

MOs diagrams of the dimer structure, obtained from theoretical calculations, show that the antibonding orbital σ*(4dz^2^) is HOMO-2. The split between the two 4dz^2^ orbitals was not enough for it to be HOMO. However, when the oligomer chain increases to n = 3, the σ*(4dz^2^) orbital becomes the HOMO in the resulting trimeric structure. Meanwhile, the LUMO, composed of π-localized orbitals from the C^N^N ligands, was stabilized through enhanced π-π stacking interactions upon oligomerization. Therefore, the HOMO–LUMO gap decreases further as the oligomer chain elongates to n = 4. Excitation of an electron from the σ*(4dz^2^) orbital (HOMO) to the ligand-localized LUMO results in a ^1^MMLCT transition. The emission wavelength of the tetramer was calculated to be a transition from the σ*(4dz^2^)(HOMO) to LUMO with a C^N^N ligand character, which is attributed to the ^3^MMLCT excited state. This contribution represented a significant advancement for the preparation of luminescent Pd(II) compounds at room temperature through the use of Pd(II)–Pd(II) interactions in aggregated states.

**Figure 8:**
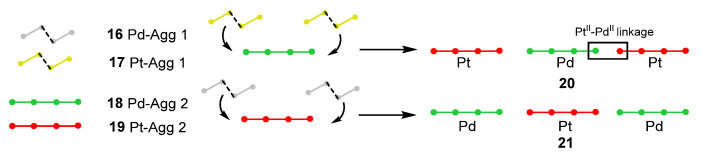
Schematic representation of the preparation of multi-block Pt(II) and Pd(II) supramolecular copolymers using a living supramolecular polymerization approach. Adapted from [67].

Che and coworkers [67] also synthesized Pd(II) and Pt(II) supramolecular copolymers **20**,**21** (Figure 8), where they observed that the copolymers made by Pd and Pt complexes through a co-assembly process are strongly phosphorescent and have distinctive photophysical properties compared to the Pd and Pt homopolymers **16**–**19**. Through a supramolecular ‘‘doping’’ approach during the co-assembly process, the frontier dz^2^ orbitals of Pd(II) and Pt(II) complexes significantly overlap, giving rise to a delocalized excited state, subsequently making the co-assemblies strongly phosphorescent due to the external heavy-atom effect from the platinum. Doping a small amount of Pt(II) complexes **17**,**19** (2 mol %) into the Pd(II) assemblies **16**,**18** significantly boosted the emission efficiency and radiative decay rate constant (Φ_em_ = 3.7%, kr = 1.83 × 10^4^ s^−1^ in Pd(II) assemblies **16**,**18**; Φ_em_ = 76.2%, kr = 58.63 x 10^4^ s^−1^ in Pt(II)–Pd(II) co-assemblies **20**,**21**), which is attributed to an external heavy-atom spin–orbital coupling effect arising from the doped Pt(II) complex with a delocalized 3[dσ*/π*] excited state.

These findings on Pt(II) and Pd(II) supramolecular copolymers with controlled sequences and greatly enhanced phosphorescence efficiencies open the door to new photofunctional and responsive luminescent metal-organic supramolecular materials, which would have wide appeal in the research on new phosphorescent materials.

**Figure 9:**
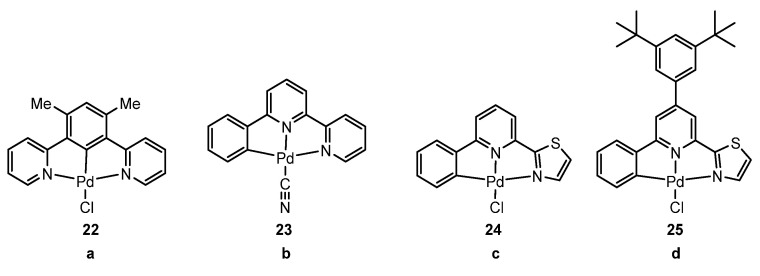
Chemical structures of Pd(II) complexes **22**–**25** containing symmetrical (**a**) and non-symmetrical (**b**,**c**,**d**) tridentate ligands, which are not luminescent in solution at room temperature, but show a strong fluorescence in solvent matrix at 77 K. Adapted from references [68,69,70].

Klein et al. have also made significant contributions during this period. In a first study [68], they investigated a Pd derivative using a dpb-type ligand **22** (Figure 9a). The Pd complex **22** was not emissive in solution at 298 K but showed a photoluminescence quantum yield of almost unity (0.98) at 77 K in a frozen glassy 2-MeTHF matrix. The authors attribute the outstanding performance of the Pd complex to the rigidity of the coordination environment and the significant ligand field splitting, which prevents radiationless deactivation processes.

In a subsequent study [69], the same authors investigated a Pd complex **23** derived from the phbpy ligand (Figure 9b). Similar to the previous case, the complex did not emit in solution at room temperature, but at 77 K in a glassy frozen DCM:MeOH matrix, it showed remarkable emission (λ_em_ = 571 nm) with a photoluminescence quantum yield of almost unity (0.98). They also studied the photophysical characteristics in the solid state. The crystal structure of such a palladium compound revealed that it forms dimeric structures with head-to-tail stacking and short metal–metal contacts. As said before, crystal packing and metal–metal interactions are important in suppressing nonradiative relaxation from metal-cantered states and achieving emission stemming from MMLCT states. At room temperature, **23** exhibits moderately intense yellow photoluminescence, showing a structureless band (λ_em_ = 561 nm, Φ_L_ = 0.14) and a lifetime of 0.35 μs. Cooling to 77 K significantly increases both the quantum yield and amplitude-weighted average emission lifetime (Φ_L_ = 0.98, τ_ampl-avg_ = 12.9 μs). This means that the radiative rate constant kr changed from 4.0 × 10^5^ to 7.6 × 10^4^ s^−1^ (a 5.3-fold decrease) on going from RT to 77 K. A substantially faster kr at 298 K likely points to the major role of TADF in the radiative decay at room temperature, whereas, at low temperature, the excited state presumably belongs to a pure triplet manifold (^3^MMLCT according to the calculations), which undergoes slower T_1_ → S_0_ relaxation. The combination of M···M interactions and increased rigidity in the solid state prevents the population of the highly distorted ^3^MC state, leading to TADF emission from a ^1^MMLCT state.

Similarly, but using a C^N^N (phen-ide)-pyridine-thiazol ligand (Figure 9c,d) [70], the authors obtained similar results to those described in the last paragraphs. Complexes **24** and **25** were non-emissive in solution at room temperature but slightly emissive at 77 K in a glassy frozen DCM/MeOH matrix (0.13 and 0.19 quantum yield, respectively).

**Figure 10:**
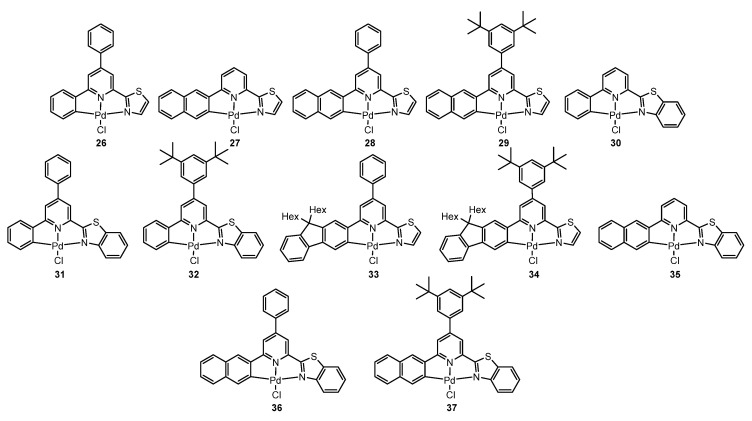
Schematic representation of the [Pd(C^N^N)Cl] complexes **26–37**. Adapted from [71].

In the same way, they used C^N^N Arylpyridine-(benzo)thiazole ligands, resulting in complexes **26**–**37**, as shown in Figure 10 [71].

Following the same trend as in the previous contributions, complexes **26**–**37** were not fluorescent at 298 K in solution. However, high photoluminescence quantum yields of up to 0.79 were recorded in frozen glassy solvent matrices of CH_2_Cl_2_ (DCM)/MeOH (1:1) at 77 K along with emission bands showing pronounced vibrational progressions and peaking at about 520 nm.

**Figure 11:**
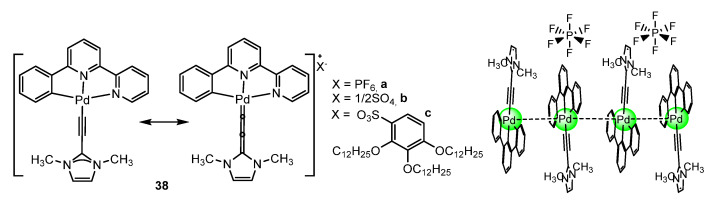
Schematic representation of the palladium(II) N-heterocyclic allenylidene complexes **38** and 1D infinite structure with a Pd(II)–Pd(II) chain with M-M interactions. Adapted from [72].

Lu et al. [72] obtained palladium(II) *N*-heterocyclic allenylidene complexes **38** with extended intercationic Pd···Pd interactions and MMLCT phosphorescence using phbpy ligand (Figure 11). The collected bulky powder samples of **38a** and **38b** were exclusively red in colour and emitted at the peak maximum of, respectively, 695 nm (τ = 1.2 ms, Φ = 43 %) and 655 nm (τ = 0.27 ms, Φ = 29 %). Compound **38c** displayed mechanochromic luminescence and vapochromic properties at room temperature. This complex could be isolated as a bright yellow solid by precipitation in methanol or as a red solid by evaporation of the toluene solution to dryness. Grinding the yellow solid in a mortar or fuming the yellow solid with toluene vapour gave a red-coloured solid. Fuming the red form with methanol vapour gave an orange-coloured solid which reversed to the red form in toluene vapour. The yellow form can only be restored by grinding the red or the orange form in methanol. At room temperature, the yellow, orange, and red forms emitted at the peak maximum of 554 nm (τ = 0.48 ms, Φ = 14%), 596 nm (τ = 0.73 ms, Φ = 20%), and 633 nm (τ =0.45 ms, Φ = 47%), respectively.

**Figure 12:**
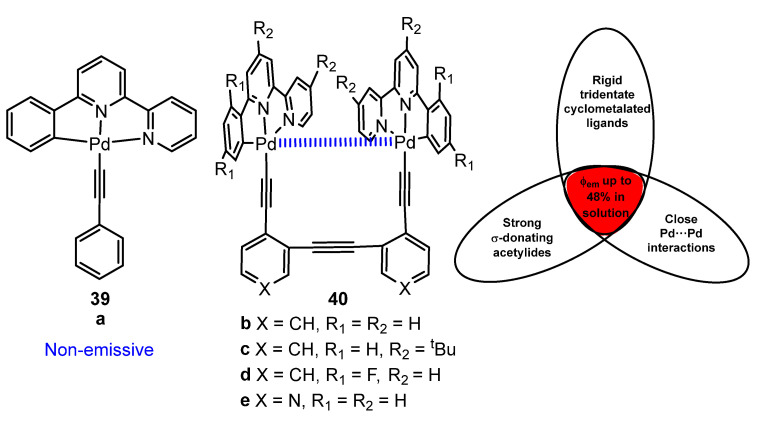
Chemical structures of pincer-cyclometalated Pd(II) arylacetylide complexes **39** and **40**. Structural features of the phosphorescent dinuclear Pd(II) **40** are highlighted in the right. Adapted from [73].

Lu and coworkers [73] reported that the dinuclear pincer-type cyclometalated Pd(II) complexes bridged by triangular diarylacetylide ligands **40**, shown in Figure 12, display unprecedentedly high phosphorescence from MMLCT excited states in diluted solutions. This molecular design combines three structural features, such as the rigid cyclometalated ligand, strong σ-donating acetylide ligands, and close intramolecular Pd⋯Pd contacts. They obtain Φ_em_ up to 48% in toluene solution at 298 K for **40c**.

**Figure 13:**
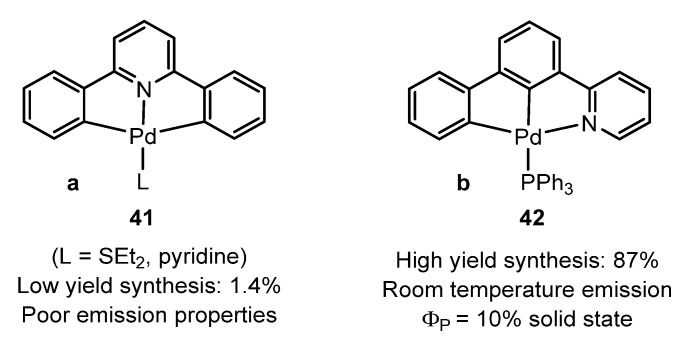
(**a**) Palindromic [(C^N^C)Pd(II)] complex **41** and (**b**) non-palindromic [(C^C^N)Pd(II)] complex **42**. Adapted from [74,75].

Breher et al. [74] obtained the phosphorescent non-palindromic [(C^C^N)-Pd] pincer complex **42**, shown in Figure 13b. In sharp contrast to the many investigations with platinum and gold complexes focused on the palindromic diphenylpyridine-based (C^N^C) pincer complexes, only one example of palladium (**41**) has been published in the literature prior to this study (Figure 13a) [75]. The non-palindromic (C^C^N) complex **42** is the first of its class using 2-([1,1′-biphenyl]-3-yl)pyridine. It exhibits intense yellow phosphorescence in solid state at room temperature (τ = 0.4 μs, Φ = 10%), but not in solution, probably due to structural distortions in the excited state. This finding highlights the unique electronic impact of the non-palindromic C^C^N-pincer ligand, as luminescence at room temperature is rare for Pd complexes.

**Figure 14:**
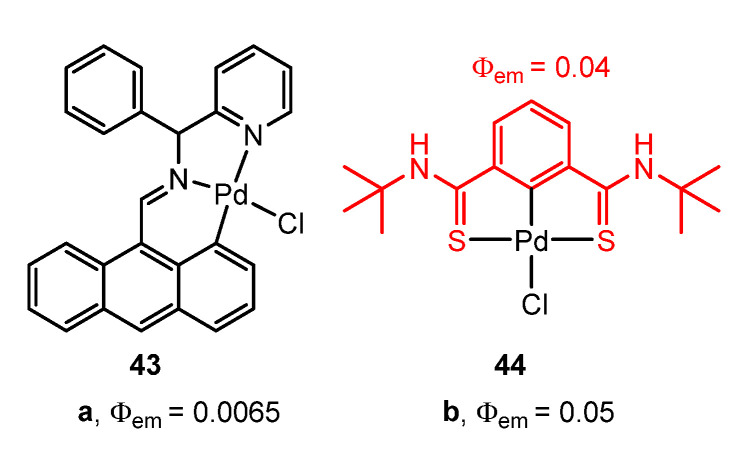
Pd(II) complexes containing tridentate ligands derived from (**a**) 2-(2(anthracen-9-ylmethylene)-1-phenylhydrazineyl)pyridine and (**b**) *N*,*N*’-di-tert-butylbenzene-1,3-dicarbothioamide by C-H activation. Adapted from [76,77].

The ligands [2-(2(anthracen-9-ylmethylene)-1-phenylhydrazineyl)pyridine] [76] (Figure 14a) and *N*,*N*’-di-tert-butylbenzene-1,3-dicarbothioamide [77] (Figure 14b) give the fluorescent orthopalladated complexes **43** and **44**, respectively, with Φ = 0.0065 in solution for the former and Φ = 0.05 in powder at room temperature for the latter, slightly higher than the value in the free ligand (4%).

### 2.4. Tetradentate Ligands

**Figure 15:**
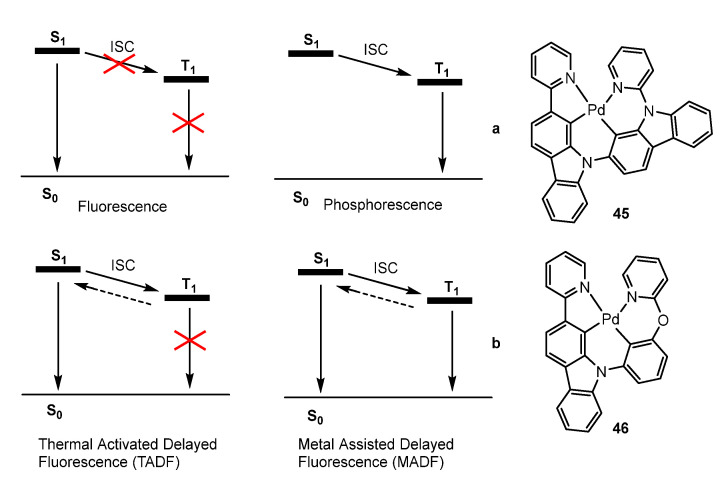
Illustration of the various emission mechanisms for organic emitters. Adapted from [78,79].

A close inspection of the works published in these last years shows clearly that tetradentate ligand scaffolds are certainly more efficient than bi- and even tridentate pincers regarding the ability to suppress vibrational relaxation pathways of photoexcited states due to their more rigid nature. In 2015, Li et al. [78] demonstrated a new mechanism for utilizing electrogenerated excitons, called metal-assisted delayed fluorescence (MADF), as shown in Figure 15 for complexes **45** and **46**. They incorporated a heavy metal ion into the complex system to achieve both efficient phosphorescence and delayed fluorescent processes, for use as efficient emitters in OLEDs.

Devices that employed complexes **45** and **46** achieved external quantum efficiencies of over 20%, and stable devices of **45** demonstrated a remarkable operational lifetime of over 20,000 h at a practical luminance of 100 cd/m^2^, with 90% initial luminance.

Cui et al. [79] conducted an extensive theoretical study on **45**. They employed both density functional theory (DFT) and time-dependent DFT (TD-DFT) methods to explore the excited-state properties of this Pd complex, which shows that the S_0_, S_1_, T_1_, and T_2_ states are involved in the luminescence. Both the S_1_ → T_1_ and S_1_ → T_2_ intersystem crossing (ISC) processes are more efficient than the S_1_ fluorescence and insensitive to temperature. However, the direct T_1_ → S_1_ and T_2_-mediated T_1_ → T_2_ → S_1_ reverse ISC (rISC) processes change significantly with temperature. At 300 K, these two processes are more efficient than the T_1_ phosphorescence and thus, enable TADF. Importantly, the T_1_ → S_1_ rISC and T_1_ phosphorescence rates are comparable at 300 K, which leads to dual emissions of TADF and room temperature phosphorescence (RTP), whereas these two channels become blocked at 100 K, resulting in only the T_1_ phosphorescence being recorded experimentally.

**Figure 16:**
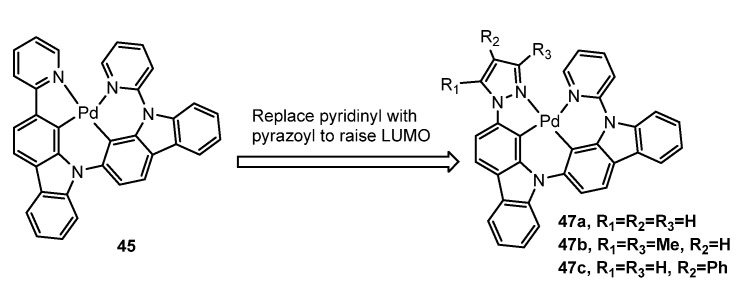
Modification of **45** for the building of blue MADF Pd(II) emitters **47a–c**. Adapted from [80].

In 2019, Li et al. [80] continued their study of metal-assisted delayed fluorescence (MADF) and Pd(II) complexes, achieving the first demonstration of the MADF process for efficient blue Pd(II) complexes. They designed a series of MADF Pd(II) complexes (**47a**,**b**,**c**) shown in Figure 16 through structural modification of **45** to achieve a blueshifted emission. An OLED device using **47b** exhibited an electroluminescence emission peak at 476 nm and a maximum external quantum efficiency (EQE) of 25.1%. The high maximum EQE of the blue OLED device indicates that both phosphorescence and delayed fluorescence are very efficient. Additionally, a stable OLED device of **47b** had a maximum EQE of 9.8% and an estimated operational lifetime of 354 h at a practical luminance of 100 cd/m^2^.

**Figure 17:**
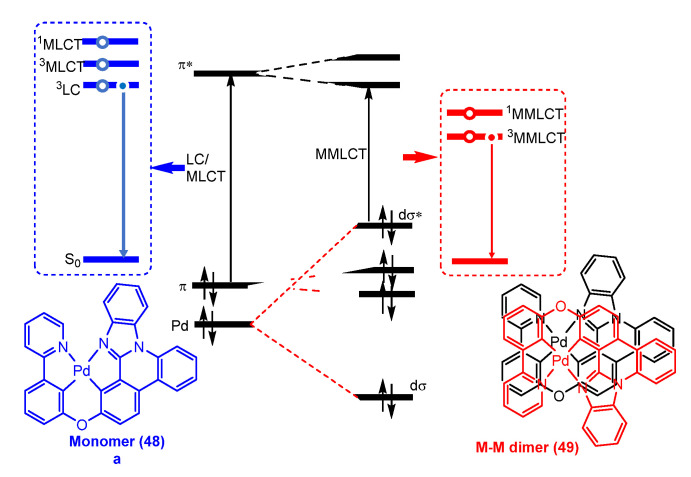
Schematic energy level diagrams for phosphorescent emitter **48**, featuring monomer **48** and M–M dimer **49** emissions. Adapted from [81].

In 2021, Li et al. [81] also obtained efficient and stable OLED devices using phosphorescent molecular aggregates of Pd tetradentate complexes. They designed and synthesized complex **48,** shown in Figure 17a, with a rigid molecular structure to enhance its chemical stability and a planar geometry to promote favourable intermolecular interactions and produce aggregate emission.

In a host-free environment, aggregates of **48** demonstrated a 100-fold reduction in luminescent lifetime (τ = 0.62 μs, while τ = 179 μs for the monomer form), close-to-unity PLQY, and predominant horizontal emitting dipoles at room temperature. Additionally, a host-free device based on **48** showed a peak EQE of 34.8% with an emission peak of 588 nm. This outstanding device performance suggests that phosphorescent molecular aggregates are potential emitter candidates for lighting and display applications. The remarkable electrochemical stability shown by these complexes in device settings, combined with the fact that Pd3O8-P has a triplet in the blue-emitting region, provides hope that the tetradentate complex design may aid in the development of efficient and long-lived blue OLEDs in the near future.

**Figure 18:**
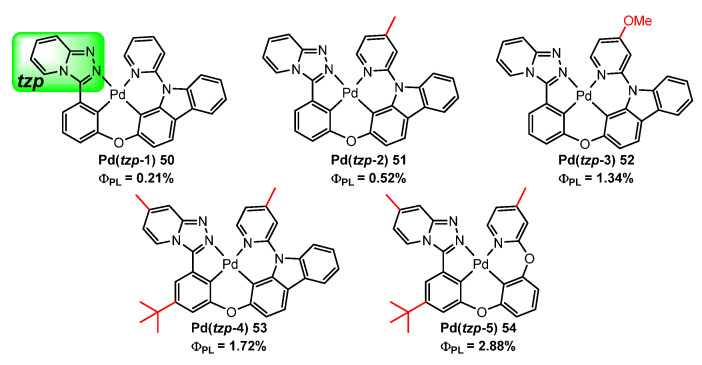
Pd(II) organometallic complexes **50–54** with tzp-type moiety. Adapted from [82].

She et al. [82] also succeeded in obtaining organopalladium compounds with tetradentate ligands that exhibit MADF **50**–**54** (Figure 18). The cyclopalladated ligand contains the structural tzpPh (3-phenyl-[1,2,4]triazolo[4,3-a]pyridine) and CzPy (carbazolylpyridine) moieties linked by an oxygen atom. The tzpPh unit works as an acceptor due to its strong electron-withdrawing character, while the carbazole group acts as a donor. On these grounds, a series of Pd(II)-based MADF materials **50**–**54** were developed. Most of the Pd(II) complexes reported here are highly emissive at 77 K in 2-MeTHF with lifetimes in the millisecond range (τ = 1.96−2.36 ms) and λ_max_ = 488−499 nm. However, the lifetimes are shortened to the microsecond range (τ = 26.7−152.9 μs in solution and 57.0−109.9 μs in thin film, respectively) at room temperature. Variations of the σ-electron-donating group R on the ligand significantly affect the photophysical properties of the complexes. In this way, the quantum efficiency of the Pd(II) complexes can be increased by more than 8-fold, as can be inferred from comparison of Pd(tzp-1) **50** and Pd(tzp-5) **54**. In addition, Pd(tzp-3) **52** has a small Δ_EST_ (0.228 eV) and exhibits strong MADF in PMMA film.

**Figure 19:**
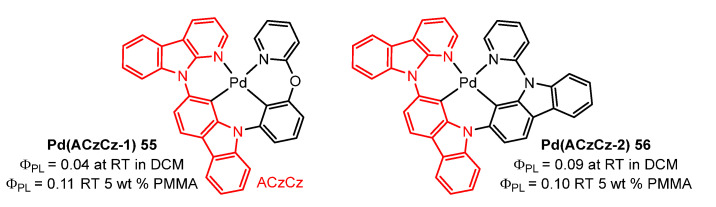
Chemical structures of ACzCz-based tetradentate Pd(II) complexes. ACz, aza carbazolyl group. Adapted from [83].

In 2021, the group of She and coworkers [83] synthesized Pd complexes **55** and **56** containing fused 6/6/6 or 6/6/5 metallocycles with azacarbazolylcarbazole-based ligands, represented in Figure 19. Both showed efficient RISC to generate MADF and phosphorescence in PMMA films at room temperature, where both ACz and py units behaved as acceptors, and CzPh and CzCz as donors. Charge transfer occurred through the central Pd ions. The Φ_PL_ of **55** and **56** were relatively low due to their weak SOC effect. Complex **55** exhibited an ultralong fluorescence lifetime of up to 1307 μs in 2-MeTHF at 77 K.

**Figure 20:**
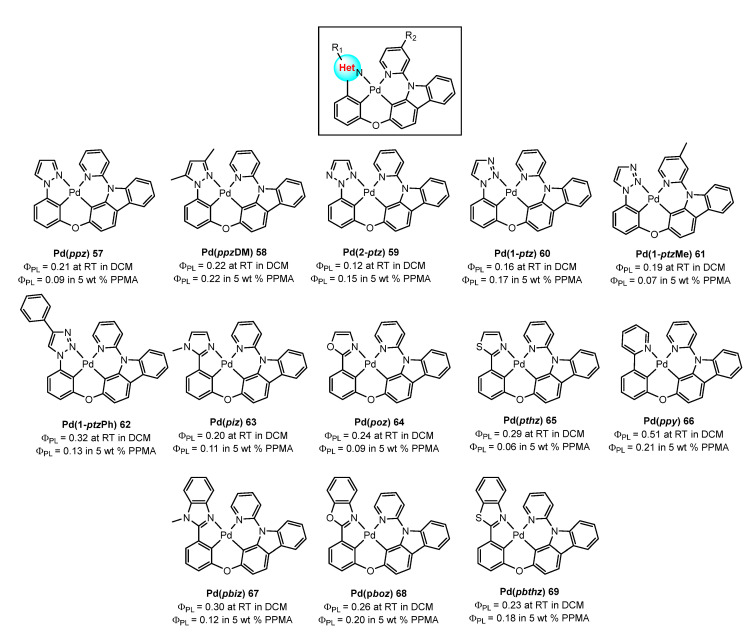
Synthesis and chemical structures of the 5/6/6 Pd(II) complexes **57**–**69** with tetradentate ligands. Adapted from [84,85].

Further work of the same group [84] also focused on the synthesis of tetradentate Pd(II) complexes **57**–**69** with deep-blue phosphorescence. The heterocyclic moiety (Figure 20) plays a crucial role in efficiently regulating the triplet-state properties. Modifying the ligands with isoelectronic imidazole **63**, oxazole **64**, and thiazole rings **65** or a pyridine ring **66** destabilizes the ^3^LE state and results in sky-blue to yellow materials with an admixture of ^3^MLCT/^3^ILCT states. Additionally, the Pd(II) complexes are strongly emissive in various matrices and achieve quantum efficiencies of up to 51%. In line with these results, tetradentate Pd(II) complexes **67**–**69** containing fused 5/6/6 metallocycles (Figure 20) from benzo[d]imidazole Pd(pbiz) **67**, benzo[d]oxazole Pd(pboz) **68**, or benzo[d]thiazole Pd(pbthz) **69** were also obtained [85].

**Figure 21:**
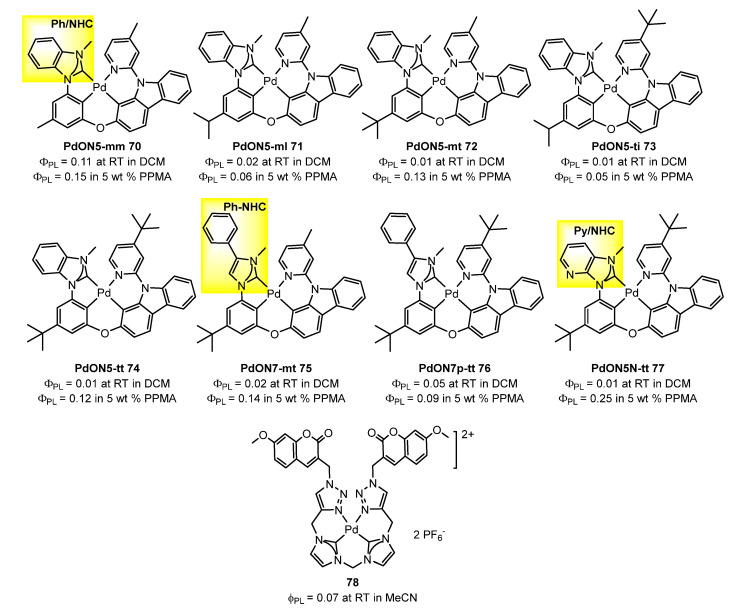
Chemical structures of Pd(II) complexes **70**–**77** with tetradentate ligands, adapted from [86], and **78**, adapted from [87].

A series of N-heterocyclic carbene (NHC)-based tetradentate Pd(II) complexes **70**–**77** (Figure 21) were designed and synthesized, employing phenylcarbene (PhNHC)-, benzocarbene (Ph/NHC)-, and pyridinocarbene (Py/NHC)-containing ligands by She at al. [86]. NHC is a very strong σ donor and a relatively weak π acceptor, which can shorten the metal-carbene bond length, shallow the LUMO energy level, and raise the d-d level of the excited state in the metal complexes. All those facts will be beneficial for the suppression of the thermally activated nonradiative decay and the enhancement of PLQY. All the NHC-based Pd(II) complexes emitted deep-blue light in 2-MeTHF at 77 K, in degassed dichloromethane solution at room temperature, and in doped PMMA film at room temperature (PLQY of up to 25% in air and a relatively short τ of 26–51 μs).

The use of ligands with NHC-type structures has also been reported by Kühn et al. [87] where a fluorescent Pd(II) complex bearing 4-methylene-7-methoxycoumarin (MMC) and 2,6-diisopropylphenyl (Dipp) substituted NHC/1,2,3-triazole hybrid ligands **78**, shown in Figure 21, was described. Complex **78** has a quantum efficiency of fluorescence of 7% in acetonitrile at room temperature.

**Figure 22:**
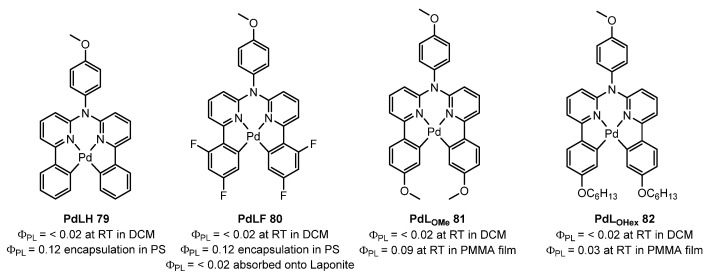
Pd(II) complexes **79–82** having a family of tetradentate ligands. Adapted from [88,89,90].

Strassert et al. [88] used a different approach to achieve Pd(II) complexes **79**,**80** bearing tetradentate luminophoric ligands with high photoluminescence quantum yields (Φ_L_) and long excited state lifetimes (τ) at room temperature. The strategy is shown in Figure 22. Incorporation of fluorine atoms into the tetradentate ligand favours aggregation, and thereby, a shortened average distance between the metal centres, which provides accessibility to metal–metal-to-ligand charge-transfer (^3^MMLCT) in **80**. Further encapsulation of **79**,**80** in 100 nm-sized aminated polystyrene nanoparticles enables concentration-controlled aggregation-enhanced dual emission. This also led to a significant enhancement of Φ_L_ and τ, suggesting a rigidification-induced suppression of non-radiative deactivation pathways. This phenomenon facilitates the tunability of the absorption and emission colours while providing a rigidified environment supporting an enhanced Φ_L_ up to about 12% and extended τ exceeding 100 µs.

The complex **80** from Figure 22 was used in a subsequent study [89] aiming an enhanced phosphorescence by adsorption onto Laponite. However, in this case, the photophysical properties were not improved by aggregation in the same way as in the previous article. Complexes **81** and **82** were further studied later [90], both showing very poor emission in fluid solutions due to dissociative MC states thermally accessible at room temperature. At 77 K, in a frozen 1:1 DCM/MeOH glassy matrix, **79**–**82** show a blue-shift in the emission spectra when compared to data gathered at 298 K, as well as unitary Φ_L_ values and a drastic increase in their lifetimes. In its crystalline form, crystals are poorly luminescent. In PMMA films, **81** and **82** show a significantly higher Φ_L_ (9% and 3%, respectively) and a longer lifetime than in Ar-purged fluid DCM at room temperature.

**Figure 23:**
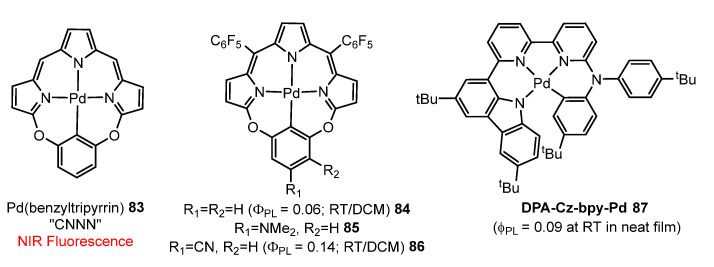
Pd(II) complexes **83–86** from tripyrrines and complex **87**, all of them showing NIR emission. Adapted from [91,92].

It has been possible also to observe fluorescence in the near-infrared (NIR) spectrum. Zhang et al. [91] have reported new tetradentate macrocyclic benzyltripyrrin (C^N^N^N) ligands. Encapsulation of the Pd(II) ion with these ligands results in complexes **83**–**86**, shown in Figure 23, which show strong NIR fluorescence (700–1000 nm) with quantum yields up to 14%. No phosphorescence is observed, which is unusual for other reported Pd-porphyrinoids (see Section 5). DFT/TDDFT calculations, along with femtosecond and nanosecond transient absorption spectroscopies, suggest that M-C bond formation leads to destabilization of the d-d excited state and less effective quenching of emission. Importantly, the small spin-orbit coupling (SOC) and the large singlet-triplet energy gap are the primary causes of the nonpopulation of triplet states. Furthermore, they demonstrated that the strong NIR emission of Pd complexes (**84** and **86**) is advantageous for in vitro or in vivo bioimaging when encapsulated into mesoporous silica nanoparticles, due to much less interference from oxygen.

Despite recent progress in palladium-based organic light-emitting diodes (OLEDs) in emission regions from blue to yellow, the corresponding Pd-based red-emissive OLEDs have not been reported yet and the related near-infrared (NIR) emission OLEDs are extremely rare. Yang et al. [92] synthesized a novel tetradentate cyclometallated palladium(II) complex **87**, shown in Figure 23, which was designed as an emissive dopant for red and NIR phosphorescent OLEDs. PLQY of 9% was measured for **87** in neat films. Optimized OLED devices using **87** exhibited peak external quantum efficiency (EQE) of 2.6% with an emission peak at 643 nm. This work presents the first example of Pd(II) complexes as red electroluminescent emitters, and sheds light on the potential of Pd(II) complexes for long-wavelength molecular emitters and electroluminescence.

## 3. Palladium Coordination Complexes

In this section, we will examine the photophysical properties of coordination compounds containing palladium where no Pd-C bonds are present. Our focus will be on how the presence of palladium affects the photophysical characteristics of the ligands, rather than on the practical applications of these compounds. We will categorize our analysis according to the type of ligands bound to the palladium centre to better understand the underlying mechanisms at play.

### 3.1. Monodentate Ligands

**Figure 24:**
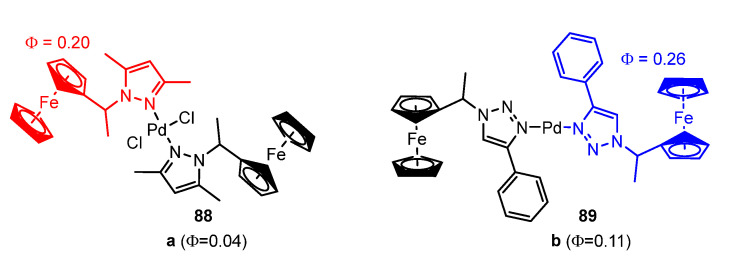
Ferrocenyl pyrazolyl (Red) and ferrocenyl triazolyl (Blue) ligands and their palladium metal complexes (**88** and **89**). Adapted from [93].

In 2020, Obuah et al. [93] explored the impact of incorporating palladium into ferrocene-based organic fluorophores, which combine the fluorescent properties of ferrocene with those of a fluorophore. It was observed that the incorporation of Pd in compounds **88** and **89**, shown in Figure 24, resulted in a decrease in both fluorescence intensity and quantum yield compared to the free ligand, this quenching due to the heavy atom effect. As shown in Figure 24, the quantum yields of the free ligands were 0.20 and 0.26, while those determined for the corresponding complexes were 0.04 (**88**) and 0.11 (**89**).

**Figure 25:**
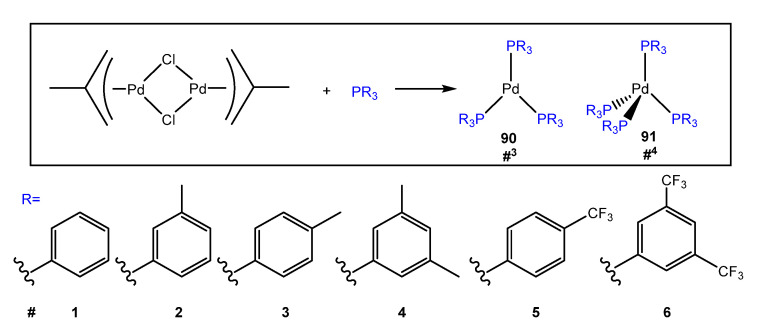
Syntheses and abbreviations of the unusual fluorescent Pd(0) complexes **90** and **91**. Adapted from [94].

An interesting example of luminescent Pd(0) compounds with phosphine ligands was reported in 2021 by Tsubomura et al. [94]. The obtained Pd(0) complexes, of stoichiometry Pd(PR_3_)_3_ **90** and Pd(PR_3_)_4_ **91** as shown in Figure 25, show high quantum yields and long lifetimes. In solid form, the emission quantum yields of these complexes range from 0.04 to 0.53, and the emitted light can range from blue to orange. In solution, three coordinated Pd(PR_3_)_3_ species **90** are the main emitting species and show orange emission. According to calculations using DFT and TD-DFT, the S_1_ and T_1_ states of Pd(PR_3_)_4_ complexes **91** are found to have higher energies than those of Pd(PR_3_)_3_ complexes **90**. Natural Transition Orbital (NTO) analysis revealed that the transitions differ between complexes **1^4^** and **1^3^**. Both have [Pd + P]-to-π* charge-transfer characteristics, but complex **1^3^** also exhibits a significant metal-centred (d→p) character. This difference in transition nature may be the main factor responsible for the observed differences in excited-state energies between the tetrakis and tris complexes. Despite the success of Pd in catalysis, the photophysical nature of Pd(0) species has not been extensively studied. This discovery may provide an opportunity for further research in this area of Pd(0) chemistry.

### 3.2. Bidentate Ligands

**Figure 26:**
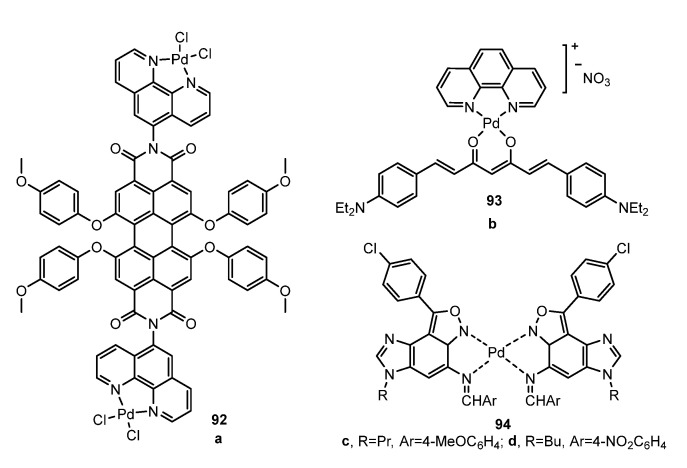
(**a**) and (**b**) Pd(II) coordination complexes **92, 93** containing 1,10-phenantroline as ancillary ligand; (**c**) and (**d**) Pd(II) coordination complexes **94** containing imines as ancillary ligands. Adapted from [95,96,97].

1,10-Phenanthroline is a classical chelating N,N-donor ligand which entails several appealing structural and chemical properties as building blocks for the synthesis of luminescent metal complexes: rigidity, planarity, aromaticity, basicity, and strong chelating capability. In 2018, Şengül et al. [95] used chelating 1,10-phenanthroline appended perylene-diimide to synthesize compound **92** (Figure 26a). The emission spectrum in DMSO showed a band at 671 nm, due to fluorescence, and blue-shifted with respect to the free ligand. It had a low quantum yield in solution (0.04), although it was slightly higher than the quantum yield of the free ligand (0.03). In 2022, de França et al. [96] synthesized the complex **93** (Figure 26b) using Curcumin dye in combination with 1,10-phenanthroline. The complex exhibits strong solvatochromism. The highest quantum yields were observed in acetone (Φ_Fl_ = 0.53), CH_2_Cl_2_ (Φ_Fl_ = 0.51), and THF (Φ_Fl_ = 0.52), due to the better stabilization of the excited state. However, once again, the incorporation of palladium did not improve the quantum yield with respect to the free ligand. Schiff bases ligands are also an important class of N,N-donor ligands due to their properties. Ramezani and Nakhaei [97] synthesized complexes **94** (Figure 26c,d) using imidazo[4′,5′:3,4]benzo[1,2-c]isoxazole-based Schiff-bases. There is also a loss of quantum yield in solution compared to free ligands (0.44 and 0.66 for free ligands and 0.29 and 0.21 for **94a** and **94b**, respectively).

**Figure 27:**
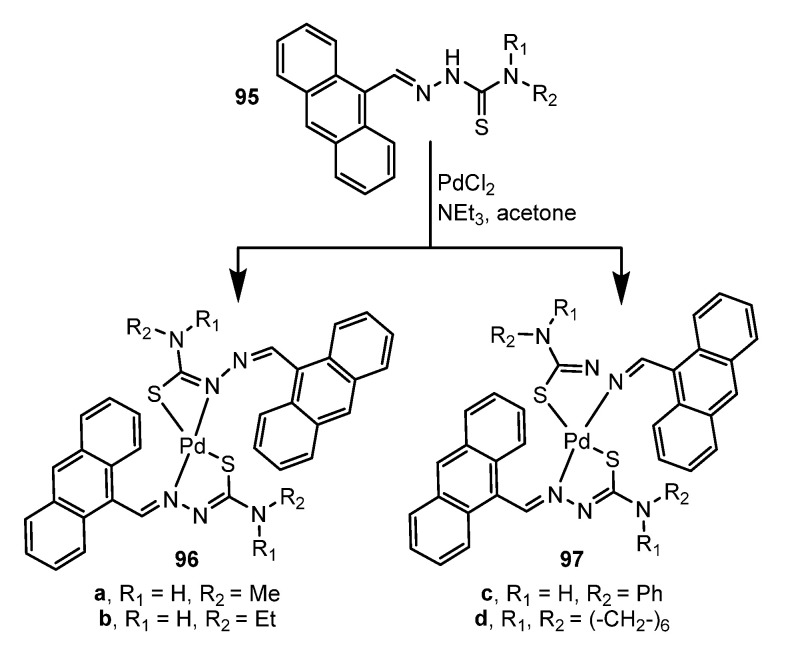
Synthesis of Pd(II) thiosemicarbazones **95–97** bearing anthracene. Adapted from [98].

Another example can be found in the use of ligands with anthracene, a linear polycyclic aromatic hydrocarbon known for its interesting photophysics and rigid geometric structure. Nguyen et al. [98] have synthesized emissive Pd(II) thiosemicarbazones bearing anthracene. The palladium complexes **95**–**97**, shown in Figure 27, are moderately emissive with quantum yields ranging from 0.10 to 0.25.

### 3.3. Tetradentate Ligands

**Figure 28:**
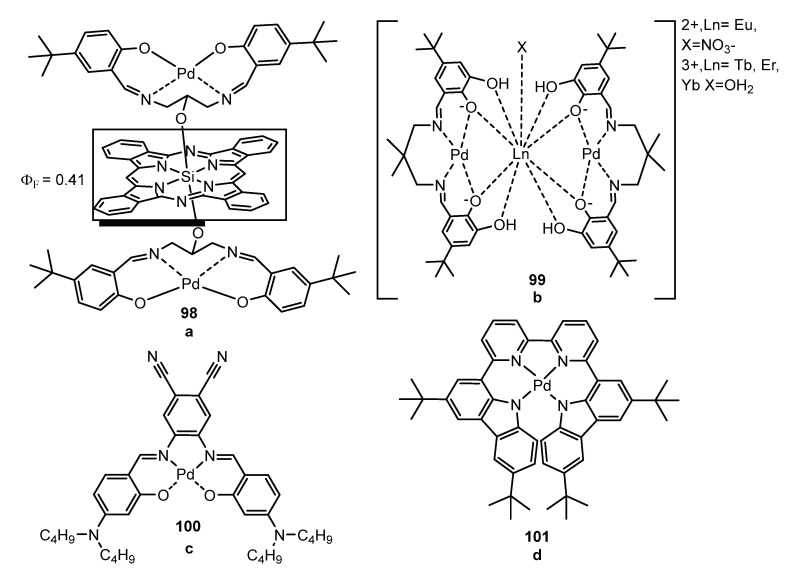
Pd(II) coordination complexes containing tetradentate Schff bases as ligands in polynuclear (**a**,**b**) and mononuclear (**c**,**d**) environments. Adapted from [92,99,100,101].

Tetradentate ligands are typically built from Schiff bases, and some representative examples of N_2_O_2_ and N_4_-donor cores are shown in Figure 28. Complexes of Pd bonded to Schiff bases have been mostly studied in polynuclear combinations with other elements such as silicon [99] (**98**, Figure 28a) or lanthanides [100] (**99**, Figure 28b), although homometallic Pd-species are also known [92,101] (**100**, **101** in Figure 28c,d, respectively). The bipyridyne-carbazole complex **101** represents the first example of a coordination complex behaving as a red electroluminescent emitter. OLED based on such a compound was fabricated with a maximum external quantum efficiency of EQE = 0.11%. The phthalocyanine-containing trinuclear Pd_2_Si-derivative **98** has a fluorescence quantum yield of 0.015 in solution. The incorporation of the Pd center to the highly luminescent silicon phthalocyanine (Φ = 0.41) produces a significant decrease in fluorescence. The trinuclear lanthanide complexes **99** schematized in Figure 28b also showed poor emissive characteristics, as well as compound **100** (Figure 28c, Φ = 0.0006 in solution at 293 K and 0.62 at 77K).

## 4. Systems Composed by Metallacages Pd_x_L_y_ and Other Supramolecular Coordination Complexes

The building of supramolecular coordination complexes (SCC) with rigid structures is nowadays another useful and efficient strategy for the obtention of Pd-based highly luminescent entities, more often than not in combination with aggregation-induced emissive motives (AIEgens) [38,39,102,103]. The restriction of intramolecular motions inside the rigid supramolecular metallacages helps for the suppression of non-radiative relaxation, allowing a remarkable increase of the quantum yield. In addition, as shown recently, the restriction of motions at a mesoscopic level can also be used to enhance the photophysical properties [36,37]. Different structures and stoichiometries have been reported, the paddlewheel Pd_2_L_4_ and the Pd_12_L_24_ nanospheres being the most common. In addition, the molecular moiety most employed to promote AIE phenomena is the tetraphenylethylene fragment (TPE) and its derivatives. This AIEgen undergoes C=C and C-C(Ph) free rotation at room temperature after excitation, opening S_1_-S_0_ deactivations due to strong vibrational couplings. Upon aggregation, these free rotations are hindered, the couplings restricted, and the deactivation partially (even totally) suppressed [75]. We want to present here how a rigid structure and the presence of AIEgens combine synergically resulting in strongly emissive compounds.

**Figure 29:**
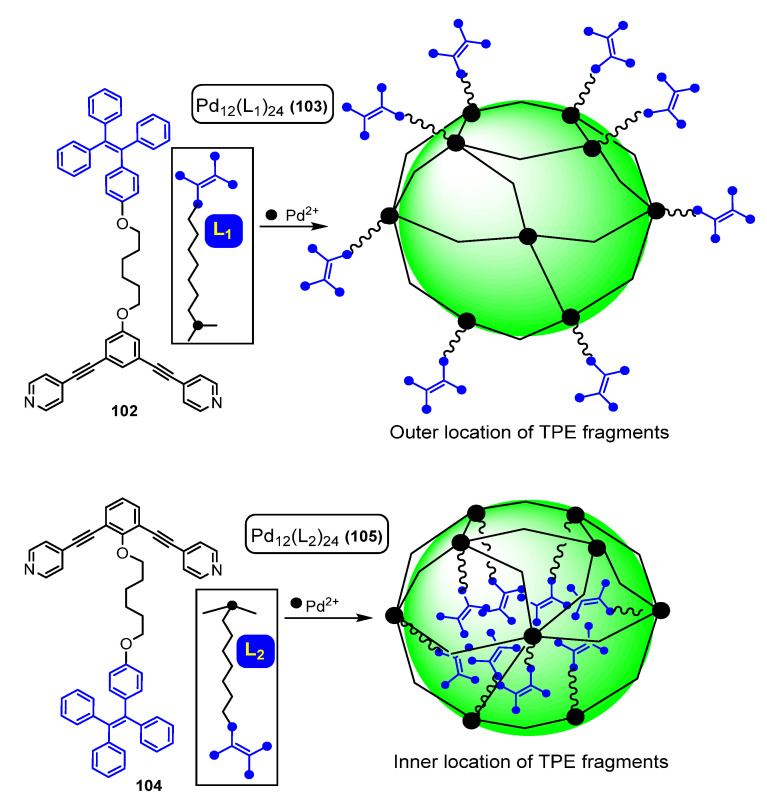
Schematic representation of complexes Pd_12_L_24_ (not all groups shown) with the bis(pyridin)-TPE derived ligands pointing towards the outer part of the Pd_12_ sphere (**up**) or towards the inner part (**down**). Adapted from [104].

Stang and coworkers have reported two different types of ligands (**102**, **104**) based on the *meta*-bis(pyridin-4-yl-ethynyl)benzene scaffold (Figure 29) [104]. One of the ligands (**102**, L_1_) contains in 5-position an ether link connected to the TPE fragment. The other ligand (**104**, L_2_), however, has the link in 2-position, that is, between the two alkynyl groups. The addition of Pd(II) or Pt(II) to **102** or **104** ligands promotes their self-assembly and the generation of the M_12_(L_1,2_)_24_(OTf)_24_ supramolecular nanospherical complexes **103** and **105**, having the same stoichiometry but remarkable structural differences, as shown in Figure 29.

For **102,** the design of the ligand puts all TPE units in the outer part of the nanosphere **103** (exo-complex), while for **104,** all TPE groups are pointing to the inner part of the cavity of **105** (endo-complex). The design of the ligands and the corresponding complexes aims to put all 24 ligands in a close environment, resulting in higher local concentrations of TPE units with restricted mobility, finally trying to reproduce a confined environment similar to that found in the β-barrel of Green Fluorescent Protein (GFP) and the corresponding increase of the fluorescence. The measurement of the photophysical properties shows that both **102** and **104** are non-emissive in solution, while complexes **103** and **105** show fluorescence enhancements. However, despite careful ligand design, these enhancements are low in Pd_12_L_24_ complexes **103** and **105** (Φ_L_ not reported), probably due to an insufficient rigidity of the nanospheres and a low deactivation of non-radiative channels [104].

**Figure 30:**
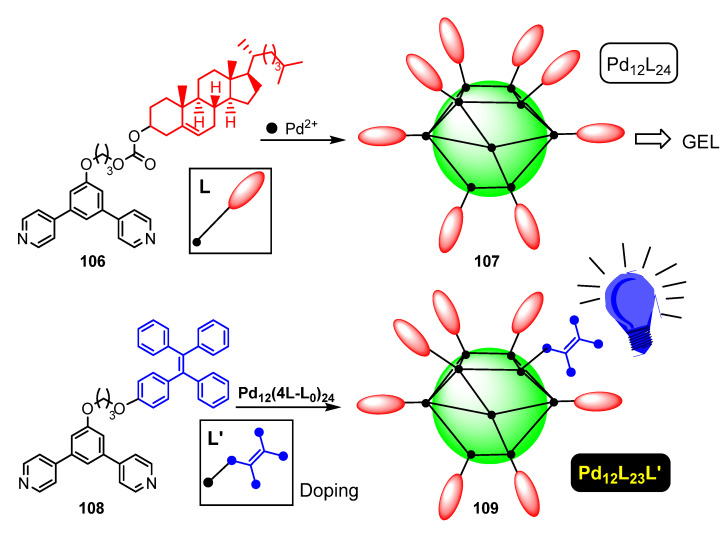
Schematic representation of complexes Pd_12_L_24_ (not all groups shown) with the bis(pyridin)-cholesteryl derived ligands (**up**) and the doped complexes showing fluorescence (**down**). Adapted from [105].

Zhang et al. have reported related Pd_12_L_24_ nanospheres **107** based on ligands derived from 1,3-di(pyridin-4-yl)benzene containing cholesteryl groups **106**, as shown in Figure 30 [105]. The main purpose of the work is the preparation of supramolecular gels via hierarchical self-organization of the cholesteryl groups on the surface of the Pd_12_L_24_ cage. In addition to an in-depth study of the gelification process, the authors go a step beyond and further functionalize the outer surface through defect engineering. Therefore, a new complex of stoichiometry Pd_12_(23L-L′)_24_ **109** could be generated via 4% doping with an L′-containing TPE ligand **108**. The study of the photophysical properties shows that **106** is emissive at 377 nm, and that the origin of this emission is the 1,3-di(pyridin-4-yl)phenyl fragment, while **108** displays emissions at 387 nm (due to the same bis-pyridylphenyl fragment) and at 433 nm due to the TPE fragment. After cage formation and doping, the corresponding Pd_12_(23L-L′)_24_ supramolecular complex **109** shows maxima of emission at 437 nm (very strong) and at 369 nm (weak), assigned to the TPE and the bis-pyridyl unities, respectively. The measurement of the quantum yields of Pd_12_L_24_ **107** and Pd_12_(23L-L′)_24_ **109** show that introduction of the doping allows an increase of Φ from 0.4% to 0.7%, due to the restricted rotation of the TPE group and the concomitant turn-on of the fluorescence.

**Figure 31:**
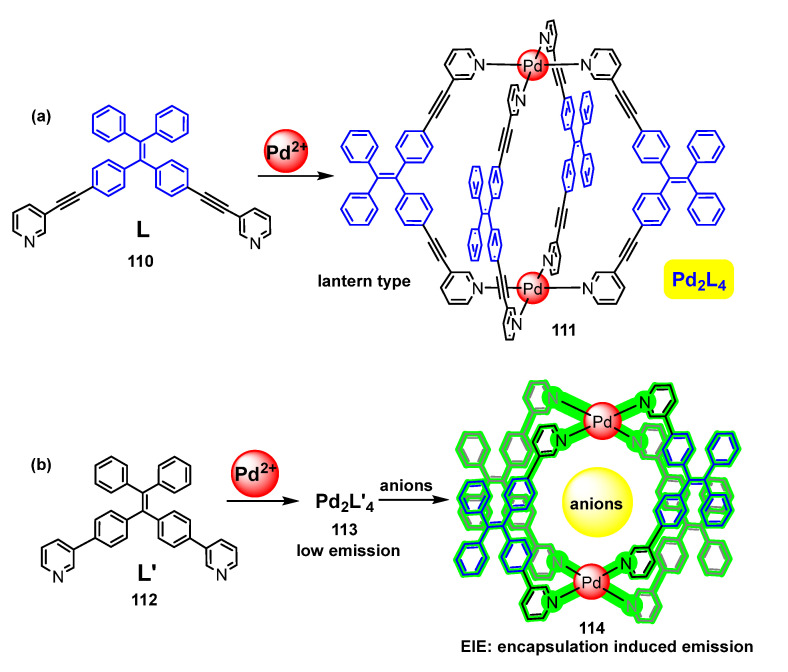
(**a**) Lantern-type complex Pd_2_L_4_ **111** built from ligand L **110** based on the TPE fragment. Adapted from [106]. (**b**) Lantern-type complexes Pd_2_L′_4_ **113, 114** built from ligand L′ **112** based on the TPE fragment: increase of the fluorescence in **114** by EIE of anions. Adapted from [107].

New lantern-type complexes of stoichiometry Pd_2_L_4_ (**111**, **113**, **114**) have been prepared from the groups of Cao [106] (Figure 31a) and Sun [107] (Figure 31b) employing standard preparative methods and ligands derived from TPE functionalized with 3-alkynylpyridyl (a, ligand L **110**) or 3-pyridyl fragments (b, ligand L′ **112**). The complex Pd_2_L_4_ **111** derived from **110** (Figure 31a) shows blue fluorescence (Φ = 1.67%) in DMSO [106]. Upon addition of water, a poor solvent for this complex, the maximum of the emission shifts to green (507 nm) and its intensity increases notably due to the AIE effect of the TPE fragment. This increment reaches a maximum when the water fraction is 60% and drops very quickly when the amount of water surpasses 70%. This is a typical behaviour for compounds showing AIE phenomena and suggests that free rotation of the C_6_H_4_-C≡C-py fragments is locked in the cage due to coordination, still allowing that of the outer Ph rings. The addition of water seems to result in the aggregation of cages and, therefore, in additional restriction of intramolecular motions with the expected increase of the fluorescence.

In the case of ligand L′ **112,** a similar lantern-type Pd_2_L′_4_ complex **113** can be obtained (Figure 31b), which shows low fluorescent properties but displays AIE. Therefore, the intensity of the fluorescence increases up to 10 times when toluene is added to a solution of the complex until the ratio toluene/DMSO is 99/1 [107]. Moreover, Pd_2_L′_4_ complex **113** can accommodate in the inner part of the palladacage different types of anions, such as HCO_3_^−^, F^−^, Cl^−^, Br^−^, I^−^, BF_4_^−^, ClO_4_^−^, and PF_6_^−^. The effect of the anion encapsulation in **114** over the fluorescence depends on the type of anion. Thus, a strong EIE enhancement is observed for HCO_3_^−^, F^−^, Cl^−^, and Br^−^, probably due to the establishment of strong H-bonds between the anions and the inner part of the cavity. Anions like BF_4_^−^, ClO_4_^−^, and PF_6_^−^ do not promote any observable effect, as a consequence of their larger size and poorer ability to form H-bonds. Finally, I^−^ show a strong quenching effect. This kind of EIE effect can find potential applications in image or biological sensing.

**Figure 32:**
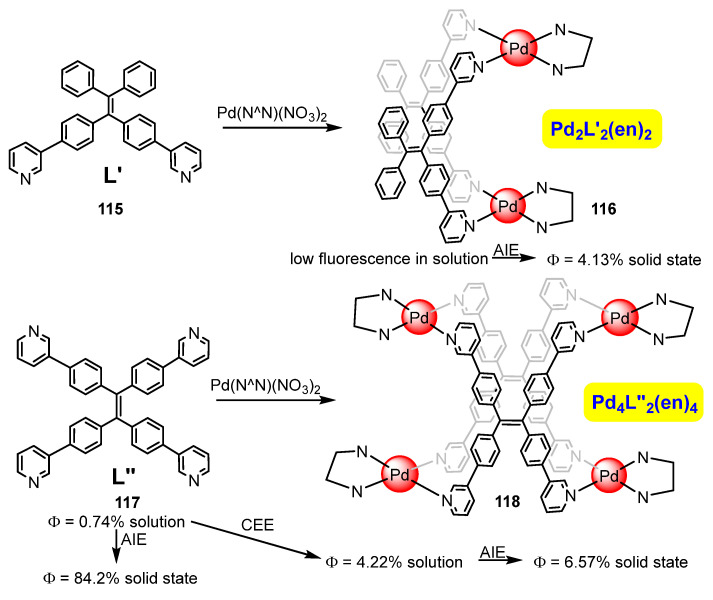
Pd_2_L′_2_(en)_2_ and Pd_4_L″_2_(en)_4_ built from polypyridine scaffolds based on the TPE fragment. Adapted from reference [108].

The group of Sun has also reported a related reactivity between *N*-multidentate ligands (**115**, **117**) and Pd(II) sources. The difference is the use of the palladium complex [Pd(en)(NO_3_)_2_] (en = ethylenediamine) as a precursor with only two vacant sites, instead of the more classical Pd(NO_3_)_2_ used in previous reports and which, in principle, has four available vacant sites [108]. The reactivity is shown in Figure 32. Reaction of L′ ligand **115** with [Pd(en)(NO_3_)_2_] affords dinuclear Pd_2_L′_2_(en)_2_ **116,** for which no enhancement of the fluorescence was observed. The reason could be related to the presence of free rotation in the outer Ph rings of the TPE fragment belonging to the complex. However, reaction of [Pd(en)(NO_3_)_2_] with L″ **117** affords tetranuclear Pd_4_L″_2_(en)_4_ **118**, for which is observed a 6-fold enhancement of the fluorescence in solution (Figure 32) due to coordination enhancement emission (CEE). Clearly, the restriction of the rotation in the four arms of L″ **117** is responsible for the observed increase. On the other hand, both the ligand L″ **117** and the corresponding complex Pd_4_L″_2_(en)_4_ **118** show AIE, much more intense in the case of the free ligand L″. The AIE is observed in solution, after water addition to DMSO solutions, and mostly in a solid state.

**Figure 33:**
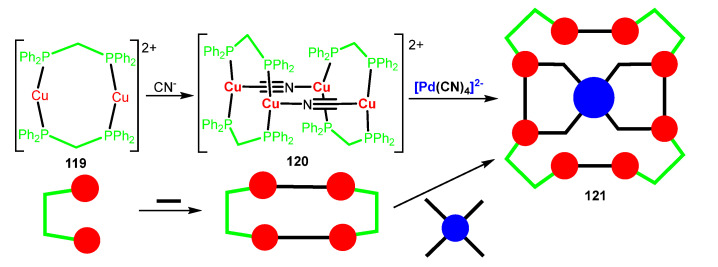
Schematic representation of nonanuclear [Cu_8_Pd(CN)_8_(dppm)_8_](PF_6_)_2_ complex **121**, built from [Cu_4_(CN)_2_(dppm)_4_](PF_6_)_2_ **120** and K_2_[Pd(CN)_4_]. Adapted from [109].

Costuas, Yam, and Lescop have reported a different approximation to the building of highly luminescent supramolecular moieties containing Pd(II) centers [109,110]. In these cases, the tetranuclear Cu(I) derivative {[Cu_2_(μ-dppm)_2_]_2_(μ-CN)_2_}(PF_6_)_2_ **120** (dppm = Ph_2_PCH_2_PPh_2_), which shows an impressive photophysical performance in solid state due to thermally activated delayed fluorescence (TADF), is used as a basic building block for the construction of higher luminescent nuclearities. As shown in Figure 33, the reaction of {[Cu_2_(μ-dppm)_2_]_2_(μ-CN)_2_}(PF_6_)_2_ **120** with K_2_[Pd(CN)_4_] gives the nonanuclear [Cu_8_Pd(CN)_8_(dppm)_8_](PF_6_)_2_ complex **121** as an air stable crystalline derivative. The same contribution also reports the synthesis of the corresponding Ni- and Pt-complexes by a reaction with K_2_[Ni(CN)_4_] and K_2_[Pt(CN)_4_], respectively, as well as a Cu-polymeric species by reaction with cyanide exclusively (not shown) [109].

The determination of the crystal structure of [Cu_8_Pd(CN)_8_(dppm)_8_](PF_6_)_2_ **121** shows that the Cu_8_Pd(CN)_8_ core is almost planar, and that the central [Pd(CN)_4_]^2−^ fragment can be considered as a template connecting four [Cu_2_(μ-dppm)_2_(CN)] moieties. This Pd derivative shows, in a solid state, a turquoise luminescence with an emission maximum at 500 nm (excitation at 350 nm) and a quantum yield of 2%. After a careful analysis of its photophysical parameters, the authors show that the emission behaviour of **121** resembles those reported for Cu(I) complexes showing TADF. Even if Pd shows a large spin-orbit coupling constant, it seems that in this case, there is no competition between the phosphorescence originated by ISC and the TADF arising from RISC. However, this is not the case for the corresponding Pt derivative, where the participation of the metal in the overall electronic transition is higher and where ISC and RISC are in competition. This exemplifies the paramount importance of the participation of the metal orbitals in the photophysical properties.

Further work showed that [Cu_8_Pd(CN)_8_(dppm)_8_](PF_6_)_2_ **121** can be obtained as different polymorphs arising from different sources [110]: (i) as minor components during the crystallization of the main component shown in Figure 33, for which the only difference is the μ^3^-bonding mode of the central cyanide ligands; (ii) mechanical treatment of the major component; (iii) thermal heating of the main component giving an irreversible melting transition; and (iv) even from DFT calculations. Not surprisingly, the polymorphs only differ in subtle details of the molecular structure, but these small are translated into huge differences in photophysical properties, and strong enhancements of the luminescence are observed [110]. As detailed previously, the quantum yield of the main component is 2%. This quantum yield increases in the minor component up to 42%, in the case of the mechanotreated sample, the increment is lower but a remarkable 27% is obtained, while for the melt sample, a quantum yield of 7% is measured [110]. The flexible coordination chemistry of Cu(I) further favours this type of remarkable behaviour.

**Figure 34:**
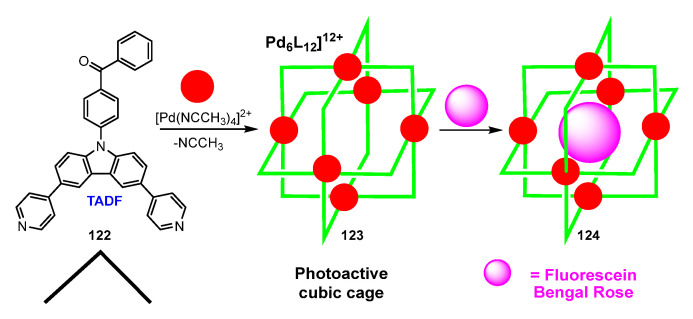
Schematic representation of the synthesis and structure of cubic cage Pd_6_L_12_ **123** and formation of host-guest complexes **124** with fluoresceine or Bengal Rose. Adapted from [111].

Zysman-Colman, Jacquemin, and coworkers have reported the synthesis of photoactive supramolecular cubic cages of stoichiometry [Pd_6_L_12_](BF_4_)_12_ **123** combining Pd^2+^ as metal center and an organic emitter **122** showing TADF, based on the carbazole core as ligand [111] (Figure 34).

The obtention in **123** of a different stoichiometry [Pd_6_L_12_] with respect to previous examples (Pd_2_L_4_ or Pd_12_L_24_, see above) resides in the angle between the two 4-pyridyl fragments, which is 93.5°. It seems that the formation of such Pd_6_L_12_ complexes is geometrically possible only when this angle has a value close to 90°. The cubic structure **123** locates the six Pd atoms at the center of each face of a cube, and each Pd is surrounded by four N,N-bridging L ligands. The complex Pd_6_L_12_ thus generated shows a quantum yield Φ_PL_ = 4% (emission at 555 nm), lower than that measured for the free ligand L **122** (52%, emission at 477 nm). The authors explain this decrease from the ligand to the cage by DFT calculations, which show that the HOMO in the cage Pd_6_L_12_ is distributed over the pyridylcarbazolyl fragments, but also that the LUMO is centered in the Pd centers and the coordinated pyridine rings. Therefore, the Pd atoms act as electron acceptor moieties and quench the fluorescence originated by TADF. Notably, the Pd_6_L_12_ cage is able to encapsulate dyes as fluorescein (up to 3 neutral molecules inside the cage in the closed lactone form) or Rose Bengal (2 dianionic molecules in the open quinoidal form) forming **124**. The authors study in detail the interactions between the cage and the hosts, showing a PeT (photoinduced electron transfer) process taking place in the case of fluoresceine; however, in the case of Rose Bengal, a photoinduced energy transfer occurs [111].

Besides the use of physical (AIE) or photophysical (TADF) strategies for the build up of luminescent SCCs and metallacages, the obvious strategy to achieve highly emissive complexes is the use of photophysically active moieties in the design of the ligand skeleton. The use of a luminescent metal would also pull together to achieve brighter emissions; however, this is not the case for palladium, as explained; therefore, we will focus on the ligands [112]. This has been probably the most employed strategy in the near past, and it is still undergoing very active research by numerous groups.

**Figure 35:**
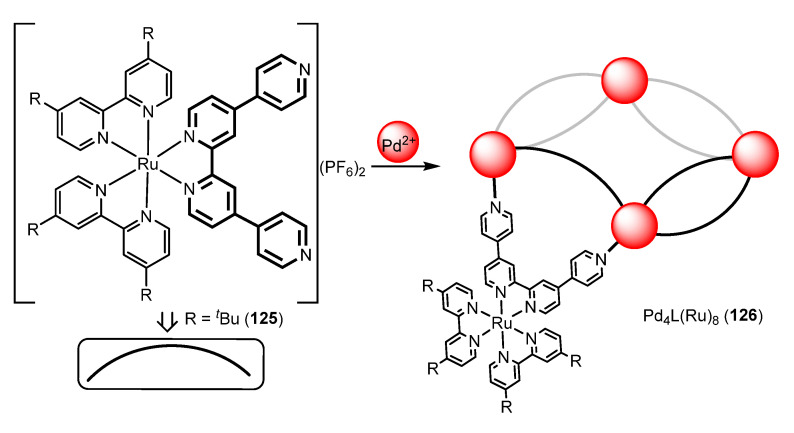
Schematic representation of the synthesis and structure of ring cage Pd_4_L_8_ **126** containing eight Ru-bipyridine derivatives **125**. Adapted from [113].

Zysman-Colman and coworkers have designed luminescent Pd_4_L_8_ cages **126**, in which each ligand L (represented by black or grey curved lines) is derived from a Ru(bipy)_2_ unit **125**, as shown in Figure 35 [113]. The structure of the complex shows that two ligands bridge two adjacent palladium centers, and that the four palladium atoms are located at the vertex of a square arrangement. This type of arrangement is possible when the angle between the donor pyridine rings is less than 90° (near 90°, the Pd_6_L_12_ structure was more favourable; see above). Actually, the angle between the two donor pyridine fragments in **125** is 69°. Complex Pd_4_L(Ru)_8_ **126** is highly emissive in CH_2_Cl_2_ solution (Φ_PL_ = 6.9%), having virtually the same quantum yield than the free ruthenium metallo-ligand **125** (Φ_PL_ = 7.3%). The analysis of the decay of the luminescence shows very similar values of the radiative and non-radiative rate constants between the free ligand L and the cage Pd_4_L(Ru)_8_. This is a fact scarcely observed, which shows that (in this case) Pd(II) does not promote the quenching of the luminescence.

**Figure 36:**
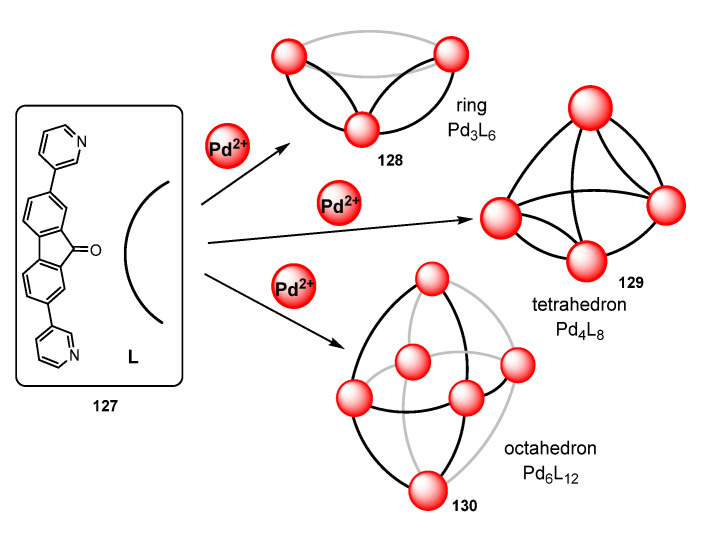
Schematic representation of the synthesis and structure of ring cages Pd_3_L_6_ **128**, Pd_4_L_8_ **129** and Pd_6_L_12_ **130** containing bridging fluorenone derivatives **127**. Adapted from [114].

**Figure 37:**
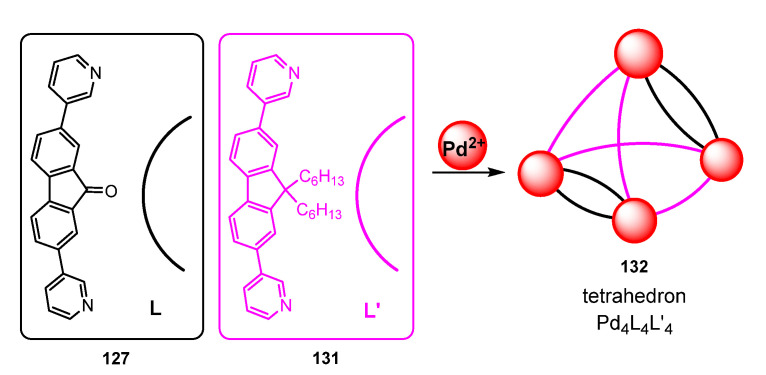
Schematic representation of the selective synthesis and structure of tetrahedral cage Pd_4_L_4_L′_4_ **132** containing two different types of ligands. Adapted from [114].

Clever et al. have reported very recently the same preservation of the luminescence is a series of very interesting polyhedral derivatives built from fluorene **131** and fluorenone **127** scaffolds [114]. The most important processes are shown in Figure 36 and Figure 37. It is necessary to highlight that the obtention of different structures resulted as being solvent dependent. Therefore, the reaction of fluorenone-based ligand L **127** (Figure 36) with Pd(II) in CD_3_CN gives a mixture of the triangular ring Pd_3_L_6_ **128**, the tetrahedron Pd_4_L_8_ **129**, and the octahedron Pd_6_L_12_ **130**, while reaction in dmso-d_6_ gives only the trinuclear derivative **128**.

Crystallization from different solvents allows one to determinate the structure of each compound separately. In the triangular ring **128,** the Pd is located at the vertices, while L ligands act bridging two adjacent Pd atoms. The structure shows that the central carbonyl can adopt two different positions, one pointing towards the outer part of the cage and the other one pointing to the close π-surface of the adjacent ligand. This proximity will be critical in order to achieve the selective synthesis of a given metallacage. In the Pd_4_L_8_ tetrahedron **129,** the Pd atoms are located at the vertices, four ligands bridge four edges, and the remaining four ligands bridge the remaining two edges. Finally, in the octahedron Pd_6_L_12_ **130,** the Pd is at the vertices and each edge is bridged by only one ligand L (Figure 36) [114].

Surprisingly, the reaction of the sterically more demanding L′ **131** (containing the bulky dihexylfluorene group) with Pd(II) gives the selective formation of the mixed tetrahedral compound Pd_4_L_4_L′_4_ **132** (Figure 37). The structure shows that it is possible to accommodate two less bulky L ligands **127** along two edges. However, the higher volume of the L′ ligand **131** determines that in the other four edges, only one ligand L′ per edge can be coordinated, due to intramolecular steric interactions. Moreover, the two hexyl groups of L′ points in all cases towards the outer part of the cage, also to minimize steric interactions. The analysis of the photophysical properties of the Pd_3_L_6_ **128** and Pd_4_L_4_L′_4_ polyhedral cages **132** show that the emissive properties of L are retained in both the trinuclear and tetranuclear complexes. Once again, the authors highlight the anomaly of the Pd-containing luminescent platforms.

**Figure 38:**
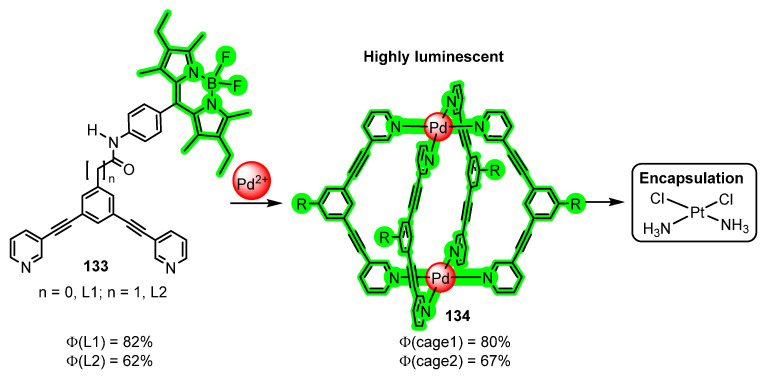
Schematic representation of the selective synthesis and structure of BODIPY—containing lantern-type cages Pd_2_L_4_ **134**, able to encapsulate cisplatin. Adapted from [115].

BODIPY is another recurrent structural moiety that is often incorporated into different ligands to confer them luminescent properties that they would not otherwise display. In a recent example, Casini et al. have reported the synthesis of two different ligands **133**, L1 and L2, shown in Figure 38, that contain the BODIPY fragment covalently bonded [115]. Using standard methods, they prepared the Pd_2_(L_1,2_)_4_ cages **134**, which resulted as strongly emissive due to the presence of the BODIPY luminophore.

For **133**-L1, the measured quantum yield in solution is 82%, while for **133**-L2, the value determined was 62%. Despite the presence of Pd in the cage, the quantum yields determined in solution for the corresponding complexes **134** are as high as 80% and 67%, respectively. The complexes thus prepared are stable in physiological conditions and also are able to encapsulate efficiently metallodrugs such as cisplatin. This ability and the notable luminescence shown by the cages prompted in-depth and successful research about their cellular uptake and distribution in different types of cancer cells by fluorescence microscopy [115]. This is an area of research undergoing a really fast growth, due to the enormous possibilities and social impact [116].

Throughout this section, we have seen the synthesis of Pd-based metallacages with more or less intense fluorescence using different approximations, such as aggregation-induced emission, restriction of intramolecular motions, thermally activated delayed fluorescence, or incorporation of fluorescent fragments. Despite the presence of palladium, a known quencher of the fluorescence, remarkable quantum yields can be obtained in the resulting cages. Besides that, the metallacages show many different applications such as the encapsulation of compounds for drug delivery purposes (as explained previously) or the generation of singlet oxygen.

In this respect, Peris et al. have reported the synthesis, characterization, and application of a metallacage of stoichiometry [Pd_4_(allyl)_4_(NHC)_4_](BF_4_)_4_, where NHC is a ligand containing two N-heterocyclic carbenes connected by a pyrene group [117,118]. This tetranuclear complex has a large inner cavity, which is able to host molecules as C_60_ or C_70_, increasing their solubility in common solvents and opening applications sometimes forbidden due to solubility limitations (for instance, light harvesting). These fullerene-containing complexes work as strong photosensitizers in the generation of singlet oxygen ^1^O_2_, which is one of the limited applications of fullerenes due to solubility issues. This singlet oxygen is further used in the oxidation of cyclic and acyclic alkenes to the corresponding endoperoxides [117,118].

In the same topic, Clever et al. have described interpenetrated double cages of stoichiometry (BF_4_)_3_Pd_4_L_8_ where the L ligands contain an acridone core and two pyridines as donor groups [119]. The cage contains three pockets, where it is possible to encapsulate different types of anions, modulating the size of each pocket in an interactive form. When small anions, like chloride, are used, it is observed that the central pocket expands and it is then able to confine six-membered rings like 1,3-cyclohexadiene or norbornadiene. In addition, the acridone-based cage (BF_4_)_3_Pd_4_L_8_ behaves as a stable photosensitizer in the generation of singlet oxygen. These two facts are combined to develop a catalytic system where the formation of endoperoxides by reaction of cyclohexadiene with ^1^O_2_ is catalysed by (BF_4_)_3_Pd_4_L_8_ or (Cl)_2_Pd_4_L_8_ cages by irradiation at 365 nm [119].

## 5. Porphyrins and Porphyrin-like Ligands

**Figure 39:**
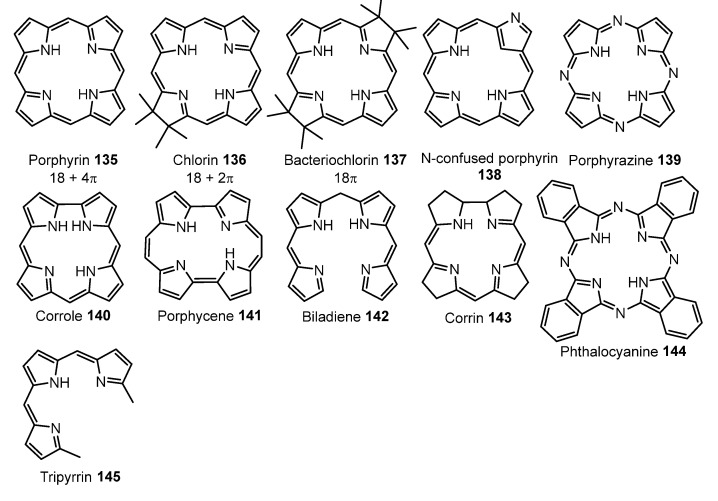
Basic structures of porphyrins and related derivatives.

Porphyrins are a large family of molecules containing a cyclic tetrapyrrole core, very stable and widely represented in the natural world (see examples **135**–**145**, Figure 39). They are natural pigments involved in very relevant biological functions, such as oxygen transport and storage (haemoglobin, myoglobin, cobalamin), light harvesting (chlorophyll) or electron transport (cytochrome P450). In fact, porphyrins are considered as the pigments of life, and certainly, life would not be as we know without the presence of porphyrins. From the chemical point of view, new porphyrins and derivatives are prepared every month focusing on new uses, and even compounds already known surprise us with unexpected applications. In this respect, porphyrins and their derivatives are employed in a large list of applications, where photodynamic therapy, theragnosis, and cancer therapy appear as the most promising and, for this reason, they are undergoing a fast development. These photo-applications are possible due to the fact that porphyrins and porphyrinoids show a high photostability and strong absorptive and emissive properties, in such a way that both fluorescence and phosphorescence can be usually observed due to efficient intersystem crossing from S_1_ to T_n_ and then internal conversion from T_n_ to T_1_. In addition, the triplet state T_1_ of porphyrins shows long lifetimes and, accordingly, porphyrins and derivatives are used as photosensitizers in the generation of singlet oxygen (^1^O_2_) and reactive oxygen species (ROS), in photo-upconversion, photo-induced charge separation, oxygen sensing, and photocatalysis [120,121,122,123,124,125,126,127,128,129,130,131]. As expected, the metallated derivatives of porphyrins (or metallaporphyrins) are known for virtually all metals from the Periodic Table, and palladium is not an exception. This fact implies an even more efficient ISC and therefore, an improvement of the phosphorescent properties [120,121,122,123,124,125,126,127,128,129,130,131]. In this section, we will focus on the most recent work developed with Pd-containing porphyrins and some related structural scaffolds.

**Figure 40:**
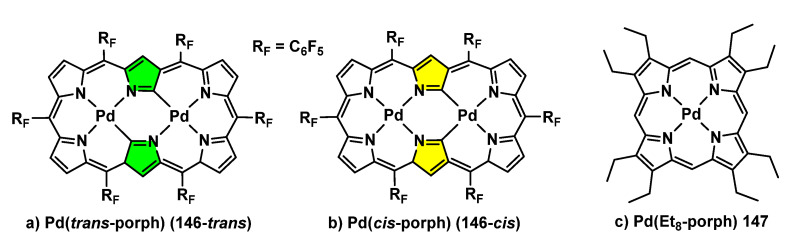
Molecular structures of palladated (**a**) **146-***trans*- and (**b**) **146***-cis*-hexaphyrines, NIR-III emitters [132]; (**c**) structure of Pd(Et_8_-porph) **147**, whose phosphorescence can be tuned to fluorescence by action of Ag-nanoprisms [133]. Adapted from [132,133].

Very few contributions dealing with fluorescence appeared during the period of time considered here. One of the most impressive has been detailed in Section 2.4 (Figure 23) and deals with the use of porphyrins in combination with orthopalladation reactions to give complexes showing near-infrared fluorescence [91]. Following in the topic of NIR emitters, the groups of Ishida, Kim and Furuta have developed molecules with absorption and emission in the low-energy NIR-III region (1500–1850 nm) [132]. This has been achieved through mixing of molecular orbitals by simultaneous metalation of a *N*-confused hexaphyrin derivative, which can be orientated in *trans*- and *cis*-forms, (**146-***trans* and **146-***cis*, respectively, Figure 40a,b) with two metals. The authors explain this mixing, taking into account the different d_π_-p_π_ interactions in the orbitals of the hexaphyrin, which results in a narrow HOMO–LUMO gap and, in general, in the possibility to modulate the orbital energies. This narrow gap is the final responsible aspect of the low-energy emission.

Both **146-***cis*- and **146-***trans*-complexes have been shown to be emissive in the NIR-III region in solution at room temperature, a situation without precedents. Upon excitation at 445 nm, **146**-*trans* in toluene solution shows an emission band at 1507 nm, this value being the lowest up to now among expanded porphyrins. However, the quantum yield of this emission is less than 0.01%, which increases at a low temperature. The emissive behaviour of **146-***cis* is quite similar. Due to these outstanding results, the potential utilization of these complexes as photoacoustic dyes has been examined. The examined complexes showed higher photoacoustic intensity peaks than the reference complex Zn(*trans*-porph), this paving the way for their use as NIR-III PA contrast agents.

A fascinating contribution comes from Sugawa and coworkers, who use the plasmon resonance of silver nanoprisms to control the emission (fluorescence and/or phosphorescence) of the Pd-porphyrin complex **147**, which otherwise only shows phosphorescence [133]. Complex Pd(Et_8_-porph) **147** shown in Figure 40c has been characterized in detail and shows an ISC efficiency of virtually 1; therefore, it shows a strong phosphorescence (Φ_phos_ = 10–30%) and an almost negligible fluorescence (Φ_fluo_ < 0.1%). These properties can be tuned, and even changed, in the presence of silver nanoprisms (AgNanoPR) of different sizes (range, 27–51 nm). The authors report how the overlapping of the localized surface plasmon (LSP) resonance of AgNanoPR with the phosphorescence band of **147** (667 nm) produces a strong enhancement of the phosphorescence of the latter. In addition, the fine tuning of the wavelength of the LSP resonance of AgNanoPR to 520 nm promotes the appearance of a new band of emission at the wavelength corresponding to the fluorescence, characterized by lifetime measurements. In addition, the distance between **147** and the AgNanoPR is critical, in such a way that the fluorescence increases as this distance decreases, while the phosphorescence increases as the distance increases [133].

**Figure 41:**
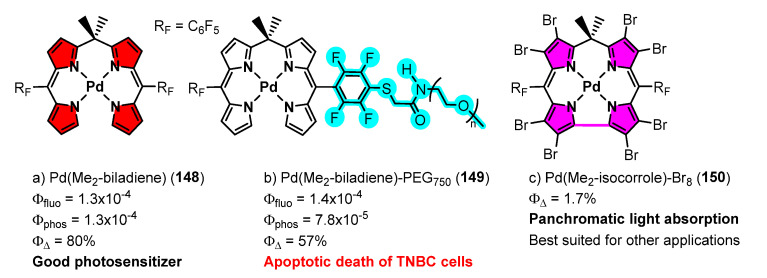
Different structural motifs based on Me_2_-biladiene and their most interesting photophysical properties. Adapted from [134,135,136].

A collection of contributions from the Rosenthal’s group reports the coordinating properties to palladium of biladienes **142** (Figure 39), open analogues of porphyrins, the electrochemical properties of the resulting complexes, and their photophysical characteristics [134,135,136,137,138,139]. As a general trend, the prepared complexes show low fluorescence but also phosphorescence, this fact prompting their study as photosensitizers for the generation of singlet oxygen (^1^O_2_) among other applications. Typical structures of the prepared complexes **148–150** are shown in Figure 41 [134,135,136,137,138,139].

The common chemical scaffold is the 10,10-dimethyl-5,15-bispentafluorophenyl-biladiene (Me_2_-biladiene), a tetrapyrrolic linear molecule where the two methyl groups at 10 position confer a high thermodynamic stability [134]. In their absence, the molecule decomposes quickly. The reaction of Me_2_-biladiene with Pd(OAc)_2_ gives the palladated derivative Pd(Me_2_-biladiene) **148** shown in Figure 41a. This molecule shows strong absorptions in the range 350–600 nm, and two clear emissions. A weak fluorescence (Φ = 1.3 × 10^−4^) is observed at 557 nm and a phosphorescence at 753 nm, for which a quantum yield of Φ = 1.3 × 10^−4^ was determined. This phosphorescence is quenched in the presence of the oxygen from the air; therefore, it has been used as photosensitizer for the ^1^O_2_ production with a quantum yield of Φ_Δ_ = 80%. The photosensitizing ability of **148** is remarkable, as can be inferred from its comparison with the free ligand (Φ_Δ_ = 1.5%). These high values of quantum yield are higher (or, at least comparable) with those reported for most of the photosensitizers used in PDT (photodynamic therapy), including currently used commercial products. The easy synthesis of Pd derivatives of biladiene, and these impressive values, open the door for the building of new PDT platforms based on biladiene metallic complexes [134]. However, Pd(Me_2_-biladiene) shows a very low solubility in water, this fact hampering its use in real physiological conditions. Therefore, Pd(Me_2_-biladiene) was functionalized to give **149** as shown in Figure 41b, incorporating a PEG chain to increase its solubility in water, allowing its study under physiological conditions [135]. The incorporation of the PEG group maintains unaltered the original photophysical properties (quantum yield of fluorescence and phosphorescence and wavelengths of emission) and still shows Φ_Δ_ = 57%. This value makes **149** worthy of being studied as a PDT agent towards MDA-MB-231 triple negative breast cancer cells. The obtained results show a high phototoxicity index, much better than those found in currently used compounds, and an efficient photoinduced apoptotic death of TNBC cells [135]. Aiming to further expand the absorption profile of the Me_2_-biladiene scaffold towards the phototherapeutic window (600–900 nm), its bromination has been reported [136]. Direct bromination of Me_2_-biladiene with *N*-bromosuccinimide (NBS) affords the corresponding perbrominated derivative Me_2_-biladiene-Br_10_ which, by reaction with Pd(OAc)_2_, gives the octabromopalladated isocorrole complex Pd(Me_2_-isocorrole)-Br_8_ **150** shown in Figure 41c. The reaction takes place through C-C bond formation between two adjacent pyrrole rings by oxidative coupling of two C-Br bonds. Complex Pd(Me_2_-isocorrole)-Br_8_ **150** is also obtained by treatment of Pd(Me_2_-biladiene) **148** with NBS, although this process affords a mixture with the hexabromo derivative Pd(Me_2_-isocorrole)-Br_6_. The resulting complexes show strong absorptions in almost the whole visible window, achieving panchromatic light absorption. Unfortunately, they do not display luminescent properties, probably due to the large number of heavy Br substituents and fast ISC processes, nor behave as photosensitizers for the production of ^1^O_2_ (Φ_Δ_ = 1%), suggesting that the triplet state has a very short halflife [136].

**Figure 42:**
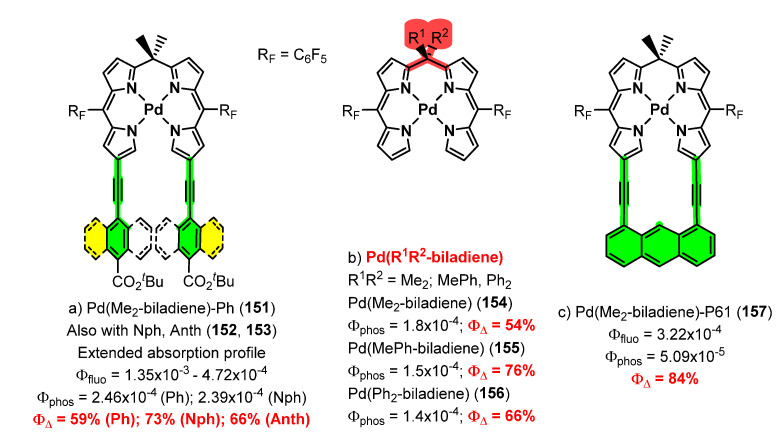
Photophysical properties and quantum yields of ^1^O_2_ sensitization for biladiene derivatives **151**–**157**. Adapted from [137,138,139].

Another useful strategy developed by the same group to expand the absorption profile of palladated biladienes towards the phototherapeutic window has been the extension of the π-system by introduction of alkynyl-aryl substituents in 2- and 18-positions of biladiene [137]. The aryl groups selected are phenyl (Ph) **151**, naphthyl (Nph) **152,** and anthracenyl (Anth) **153**, and the complexes obtained following a classical Sonogashira coupling are represented in Figure 42a. As expected, the absorption maxima of the three complexes were red-shifted with respect to the parent compound Pd(Me_2_-biladiene) **148**. In addition, the complexes showed emission profiles also red-shifted, but present some differences between them. In this respect, the phenyl- and naphthyl derivatives [Pd(Me_2_-biladiene)-Ph **151** and Pd(Me_2_-biladiene)-Nph **152**] showed both fluorescence and phosphorescence bands, the latter disappearing in the presence of oxygen, while for the anthracenyl derivative **153,** there was no phosphorescence band (Figure 42a). Despite this fact, the three complexes show promising photochemical properties by excitation in the phototherapeutic window (600–900 nm), because after excitation at a wavelength higher than 650 nm, all of them photosensitize the obtention of ^1^O_2_ with quantum yields as high as 59% (**151**, Ph), 73% (**152**, Nph), and 66% (**153**, Anthr) [137]. In a subsequent contribution, the effect of the change of the substituents at the *meso*-10 position of the biladiene skeleton has also been examined [138]. All complexes mentioned up to now contained two methyl groups at such a position (**154**), while in this work, complexes with two phenyl rings (**156**), or with one methyl and one phenyl (**155**), were prepared and studied (Figure 42b). Surprisingly, the absorption profiles increase as the number of Ph rings at this position increases and, moreover, the ability to photosensitize the formation of ^1^O_2_ follows the same trend [138]. The in-depth analysis of the excited states dynamics shows that the ISC quantum yields change as a function of the meso-C substituents, and that the tendencies observed in ISC efficiency fit with those observed for sensitization. A last example aiming to expand the π-system of the biladiene is shown in Figure 42c [139]. The design of a compound called Pd(Me_2_-biladiene)-P61 **157** obeys the combination of two factors: the red-shift of absorptions and emissions due to the π-extension achieved introducing alkynyl groups at 2- and 18-positions, and the additional extension of π-conjugation by coupling an anthracene moiety with the two alkynyl fragments, closing the circle. In this way, the delocalization and π-conjugation encompass the whole molecule. From the point of view of the ^1^O_2_ production, this complex outperforms all other compounds reported up to now, because the quantum yield measured is as high as 84% irradiating at 600 nm. Both facts (photosensitization quantum yield and absorption in the therapeutic window) point to a promising future for this type of compound as PDT cancer agents.

**Figure 43:**
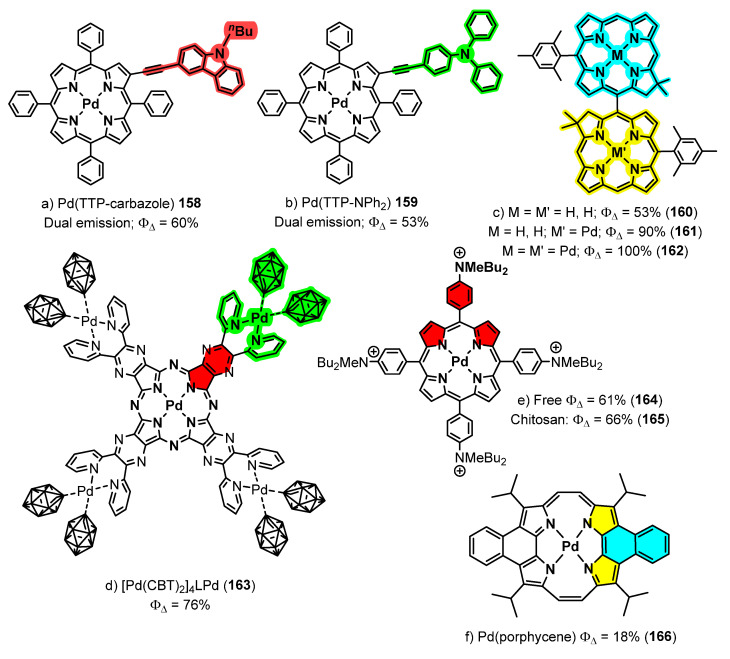
Phorphyrin- and porphyrin-like-containing complexes as photosensitizers for the ^1^O_2_ production. Adapted from [140,141,142,143,144,145].

As it is possible to infer from previous paragraphs, the research in metallic complexes of porphyrins and derivatives is a very active area. New examples of sophisticated systems have appeared in the literature about the use of pallada-porphyrins as photosensitizers for the production of singlet oxygen and its application to photodynamic therapy. In all reported cases, the complexes show weak fluorescence and phosphorescence, the latter band being quenched in the presence of oxygen. Figure 43 presents the most remarkable contributions reported from 2018 up to now. Gupta and coworkers prepared the dyads **158** and **159** shown in Figure 43a,b, where Pd(TPP) complex has been modified by the introduction of alkynyl-carbazole and -diphenylamine moieties (TPP = tetraphenylporphyrin). These dyads show remarkable quantum yields of 60% and 53% for the singlet oxygen photosensitization reaction [140] and, therefore, could be used as photocatalysts in PDT and in oxidation reactions. The group of Ptaszek reported dyads composed of two different chlorin fragments **160–162**, such as those shown in Figure 43c, which show outstanding photophysical properties tunable as a function of the metals incorporated in M and M′ positions [141]. The uncomplexed form **160** (M = M′ = H,H) shows a remarkable value of 53% for the photosensitization to produce ^1^O_2_. Interestingly, the incorporation of Pd to the chlorin scaffolds enhances the quantum yield of such sensitization: the first incorporation (M = H,H; M′ = Pd **161** in Figure 43c) gives a value of 90% (1.7 fold increase), while the introduction of the second Pd center in **162** allows a quantum yield of 100% to be reached [141]. Porphyrazine derivatives (N-versions of phthalocyanines) have also been prepared and characterized by Donzello et al. [142]. The pentanuclear palladium derivative **163** shown in Figure 43d behaves as a strong photosensitizer for ^1^O_2_ production, exhibiting a quantum yield (76%) higher than those reported for similar porphyrazine complexes (40–60%). In addition to their classical use in PDT, these compounds could be used in boron neutron capture therapy as bimodal anticancer drugs. The groups of Sen and Nyokong have reported very recently the immobilization of functionalized Pd(TPP) complexes in chitosan. In the functionalized TPP **164**–**165**, the peripheral phenyl rings contain dibutylamino or (methyl)(dibutyl)ammonium groups, as shown in Figure 43e [143]. As is usual in this type of complex, they show very low quantum yields for fluorescence, indicating the effective population of the triplet excited state, and high quantum yields for the singlet oxygen sensitization, which in this case increases after immobilization. In this case, the authors propose the use of the immobilized compounds in photodynamic antimicrobial therapy [143].

Palladium complexes **166** from dinaphthoporphycene have been reported by Panda et al. [144]. Porphycenes are well known as they show superior ability as photosensitizers for singlet oxygen production in comparison with the corresponding porphyrin derivatives. In this case, neither the free ligand nor its out-of-plane complexes show any capability to generate ^1^O_2_. Surprisingly, the Pd complex **166** from dinaphthoporphycene (Figure 43f) was able to photosensitize the production of singlet oxygen although with a modest 18% quantum yield. Finally, emitters in the NIR region (670–770 nm) [145] based on palladium complexes with phosphorylated porphyrins have been reported by Borisov, Gorbunova, and Bessmertnykh-Lemeune. A series of porphyrins with different degrees of substitution, containing phosphorylated groups and other substituents of different nature in a variety of positions, is studied in detail. The obtained results show that all complexes are phosphorescent and that the presence of the electron-withdrawing phosphorylated moieties improves the photophysical parameters of the porphyrin. Therefore, complexes have a good photostability and values of Φ_Δ_ in the range 3.4 to 5.8% were obtained, being good candidates to be used in oxygen biosensing.

**Figure 44:**
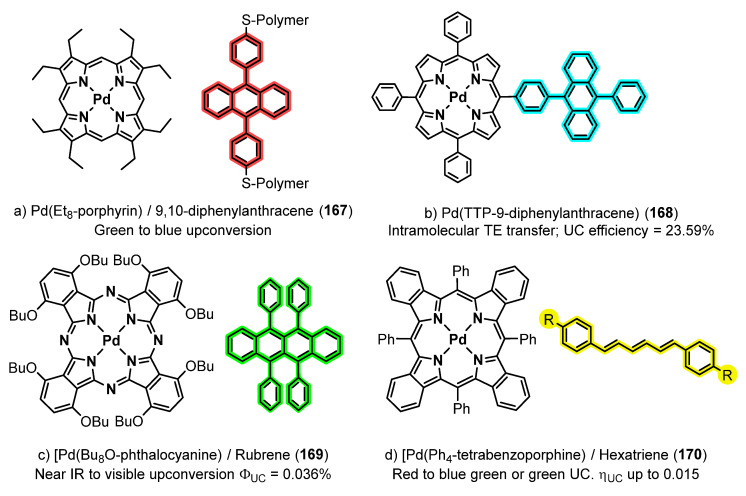
Representative examples of Pd-porphyrin complexes as photosensitizers for upconversion in organic substrates. Adapted from [146,147,148,149,150,151].

We cannot finish this section without mentioning the fact that Pd complexes of porphyrins, and related species, are renowned photosensitizers for the process of light upconversion (UC) by triplet–triplet annihilation (TTA) [146,147,148,149,150,151]. This process involves the participation of two different molecules, the photosensitizer and the acceptor. The photosensitizer harvests the light and is excited to its triplet state, which reacts with the ground state of the acceptor through a triplet–triplet energy transfer process resulting in the formation of the triplet state of the acceptor. The collision of two molecules of the acceptor in their triplet state results in the formation of a molecule of acceptor in the ground state S_0_ and another one in the first singlet excited state S_1_. This singlet S_1_ state, in the absence of non-radiative processes, will emit one photon of higher energy than that of the photon employed to excite the photosensitizer. This is, briefly, the basic idea of the upconversion, which has a large list of applications in photovoltaics, OLEDs, image, photocatalysis, and so on. As stated above, Pd complexes containing porphyrins as ligands **167**–**170** (examples collected in Figure 44) are good photosensitizers for upconversion, and relevant contributions have appeared in recent years using 9,10-diphenylanthracene **167** [146,148,151], rubrene **169** [147,149,151], diphenylhexatriene **170** [150], and perylene [151], among others, as acceptor molecules.

## 6. Conclusions

Throughout this contribution, we have presented how the incorporation of palladium metal to organic ligands can have a positive effect over their photophysical parameters, giving complexes with fascinating properties and applications: use in OLEDs, bioimage, photodynamic therapy, or upconversion. The deleterious effects of palladium as a heavy atom, manifested through the spin–orbit coupling constant, can be circumvented by molecular design engineering. Therefore, the introduction of high-field ligands makes it possible to increase the HOMO–LUMO gap and deactivate the relaxation pathways that take place through the d orbitals of the metal. In the same way, the increase in the denticity of the ligands and the rigidity imposed by multidentate scaffolds suppresses most of the molecular motions responsible for non-radiative deactivations. To these well-known phenomena, new fluorescence amplification tools have been added the past years based on new physical phenomena, such as the aggregation-induced emission, the metal (thermally)-activated delayed fluorescence, and the upconversion by triplet–triplet annihilation. The use of these new tools is allowing a fast development and providing access to applications that were unimaginable just a few years ago, both in the development of new materials for optics and energy and in the area of diagnosis and treatment of diseases such as cancer. For all these reasons, it is not unreasonable to think that despite being an odd couple, luminescence and palladium allow us to glimpse high-interest applications in the near future and have a long way to go.

## Data Availability

No new experimental data have been generated on this work.

## Abbreviations

ACQ: aggregation caused quenching; AIE: aggregation induced emission; CDS: coordination driven supramolecular compounds; CEE: coordination enhancement emission; DCM: dichloromethane; DMSO: dimethylsulfoxide; EIE: encapsulation induced emission; EQE: external quantum efficiency; HOMO: highest occupied molecular orbital; ILCT: intraligand charge transfer; ISC: intersystem crossing; LUMO: lowest unoccupied molecular orbital; MADF: metal assisted delayed fluorescence; MC: metal centered; MLCT: metal-to-ligand charge transfer; MMLCT: metal-metal-to-ligand charge transfer; NHC: N-heterocyclic carbene; OLED: organic light emitting diode; PDT: photodynamic therapy; PEG: poly(ethylenglycol); PeT: photoinduced electron transfer; PLQY: photoluminescence quantum yield; PMMA: poly-methylmethacrylate; RIM: restriction of intramolecular motions; rISC: reverse Intersystem crossing; SCC: supramolecular coordination compounds; SOC: spin-orbit coupling; TADF: thermally activated delayed fluorescence; THF: tetrahydrofurane; TPE: tetraphenylethylene; TPP: tetraphenylporphyrin; TTA: triplet-triplet annihilation; UC: upconversion.
